# Performance of the neutrophil-to-lymphocyte ratio as a prognostic tool for survival in solid cancers

**DOI:** 10.3389/fonc.2025.1616477

**Published:** 2025-07-21

**Authors:** Irene Carrión-Barberà, Christian Lood

**Affiliations:** Division of Rheumatology, University of Washington, Seattle, WA, United States

**Keywords:** survival, prognosis, biomarkers, blood cell components, cancer

## Abstract

Neutrophils and lymphocytes are crucial players in cancer progression, with the neutrophil-to-lymphocyte ratio (NLR) emerging as a potential prognostic biomarker. However, its clinical relevance remains uncertain. This study retrospectively analyzed individual patient data from five Phase III clinical trials encompassing multiple cancer types to assess the prognostic value of baseline neutrophil (N1), lymphocyte (L1), and NLR (NLR1) counts for overall survival (OS) and progression-free survival (PFS). Survival outcomes were evaluated using Kaplan-Meier analyses, Cox proportional hazards models, and receiver operating characteristic curves, with subgroup analyses conducted across demographic and clinical subpopulations. High NLR1 and N1 and low L1 were associated with worse OS and PFS. In Cox uni- and multivariate analyses, NLR1 was an independent predictor of OS (HR: 1.508 (95% CI: 1.390 – 1.636, p<0.001)), while N1 and L1 were only significant when analyzed categorically (N1 HR: 1.390, L1 HR: 0.801; all p < 0.001). Similar patterns were observed for PFS (NLR1 HR: 1.261, N1 HR: 1.154, L1 HR: 0.848; all p < 0.001). Biomarkers showed higher HR in < 60 years, Non-White, Stage IV, and Eastern Cooperative Oncology Group Performance Status = 1 patients. Kaplan-Meier analysis confirmed worse survival for most patients with highest NLR1 or N1 and low L1 and low L. These findings confirm the prognostic role of blood cell components in cancer risk assessment and underscore the importance of personalized biomarker-based stratification, warranting further prospective studies to establish standardized clinical use.

## 1 Introduction

Neutrophils play a role in various stages of cancer development and progression through multiple mechanisms. They contribute to carcinogenesis by inducing DNA damage and mutations via reactive oxygen species (ROS), nitric oxide (NO), microRNAs, and matrix metalloproteinase 9 (MMP9). Additionally, they promote immunosuppression by releasing arginase-1 (Arg-1), which inhibits T cell activation and proliferation. Neutrophils also facilitate tumor progression by eliminating senescent cells through IL-1RA and enhance cancer growth by producing cytokines and neutrophil extracellular traps (NETs), which create an acidic microenvironment that supports tumor proliferation. Furthermore, they also drive metastasis by promoting angiogenesis, cancer cell migration, intravasation, survival in circulation, and extravasation. NETs also reactivate dormant cancer cells, contributing to metastatic expansion (1). Some studies have linked elevated neutrophil counts with poor prognosis across various cancers, including colorectal cancer (2) and melanoma (3). However, most of the studies and the available systematic reviews on the topic focus on the role of tumor-associated neutrophils (TAN) (4) and not on neutrophil counts in peripheral blood. Even on the association of TAN with cancer prognosis the evidence is scarce, with the most recent systematic review conducted in 2020 finding that much of the available evidence was weak or uncertain (5) and that, despite the strong mechanistic plausibility, further research was needed.

The association between low lymphocyte counts and cancer prognosis is also multifaceted. One proposed mechanism involves impaired immune surveillance, where reduced lymphocyte levels fail to effectively control tumor cell proliferation (6). Additionally, tumor-induced lymphocyte apoptosis and disrupted lymphocyte homeostasis contribute to lymphopenia. Tumors can promote apoptosis by producing pro-apoptotic ligands such as FasL and TNFβ, which reduce circulating lymphocytes. Furthermore, cytokines released by the tumor microenvironment may impair dendritic cell differentiation and function, further weakening immune responses (6). Somes studies have demonstrated the prognostic relevance of low lymphocyte counts in cancer such as the one by Ray-Coquard et al. which identified lymphopenia as an independent prognostic factor for progression-free survival (PFS) in multiple solid and hematologic malignancies (7).

The neutrophil-to-lymphocyte ratio (NLR) is a novel marker related to inflammation and stress that has received a lot of attention lately as an easily accessible, low-invasive, routinely-determined biomarker in potentially any condition that is associated with a systemic inflammatory response, among them coronary heart disease (8), infections (9), autoimmune diseases (10), psychiatric disorders (11) and cancer (12). Calculated just by the ratio between the count of neutrophils and lymphocytes in peripheral blood it is an expression of the relationship between the innate immune system (through neutrophils) and the adaptative one (driven by lymphocytes) (13).

NLR has been associated with higher overall mortality in the general population, as well as with specific causes of mortality (13). Particularly in cancer, multiple works have tried to elucidate the prognostic role of NLR in different characteristics of the disease over the last years, in their effort to find a robust prognostic biomarker that can help stratify patients and offer them the best possible personalized therapeutic intervention. Although evidence gathered through metanalyses seems to indicate that NLR is associated with poor outcomes in patients with solid tumors, several hindrances limit the ability to draw conclusions and probably explain why NLR remains underutilized in clinical practice (5, 14, 15). First, NLR is affected by multiple factors such as age, race, anti-inflammatory medications such as corticoids and concomitant chronic diseases (16). These confounders reduce its specificity and may obscure its true prognostic value. Second, determining an optimal NLR cut-off value remains challenging, as studies report varying thresholds based on demographic and tumor characteristics. This raises the question of whether NLR’s prognostic value could be improved in certain patient subsets. In light of the variation observed in meta-regressions of NLR cut-off values and effect size in the umbrella review by Cupp et al., where very few associations reached statistical significance, more studies that account for confounding factors are needed to clarify if the association is truly significant and to standardize cut-off values (5). The absence of prospective validation in large trials, the perception that NLR lacks mechanistic insight compared to molecular or genomic biomarkers as well as the growing emphasis on precision oncology may also contribute to this underuse, as clinicians often prioritize biomarkers with direct therapeutic implications. Finally, the heterogeneity of existing studies, their variable methodological quality, and small-study effects in meta-analyses further limit the reliability of conclusions, highlighting the need for prospective studies that integrate clinical context, standardize analytical approaches, and assess complementary biomarkers to improve clinical adoption of NLR (5).

Second, determining an optimal NLR cut-off value remains challenging, as studies report varying thresholds based on demographic and tumor characteristics. This raises the question of whether NLR’s prognostic value could be improved in certain patient subsets. In light of the variation observed in meta-regressions of NLR cut-off values and effect size in the umbrella review by Cupp et al., where very few associations reached statistical significance, more studies that account for confounding factors are needed to clarify if the association is truly significant and to standardize cut-off values (5). Finally, the heterogeneity of the studies, their varied quality and small-study effects when performing metanalyses limit the reliability of conclusions, which warrants further studies to overcome these limitations (5).

With the intention of adding to the available current evidence to help validate any of these blood cell components as biomarkers for risk stratification, we conducted an in-depth analysis of the association of NLR, neutrophil and lymphocyte absolute counts and the changes of NLR over time with survival outcomes. We tried to define the optimal group of patients in whom they have the best prognosis performance, as well as its optimal cut-off values. We evaluated individual data from a large retrospective cohort formed by 5 different phase III cancer clinical trials around the world and assessed the biomarkers in relation to survival measures on each of the specific strata of patients.

## 2 Methodology

### 2.1 Study design and inclusion criteria

Retrospective study of patients included in 5 different phase-III clinical trials, each of them with their own inclusion and exclusion criteria, detailed in the paper where the individual results of each trial were reported. The studies are the following:

1. A Phase 3, Double-Blind, Placebo-Controlled Study of Maintenance Pemetrexed plus Best Supportive Care *vs*. Best Supportive Care Immediately Following Induction Treatment with Pemetrexed + Cisplatin for Advanced Nonsquamous Non-Small Cell Lung Cancer (17).
2. A Randomized, Double-Blind, Phase 3 Study of Docetaxel and Ramucirumab *vs*. Docetaxel and Placebo in the Treatment of Stage IV Non-Small Cell Lung Cancer Following Disease Progression after One Prior Platinum-Based Therapy (18).
3. A Randomized, Double-Blind, Multicenter Phase 3 Study of Irinotecan, Folinic Acid, and 5-Fluorouracil (FOLFIRI) Plus Ramucirumab or Placebo in Patients With Metastatic Colorectal Carcinoma Progressive During or Following First-Line Combination Therapy With Bevacizumab, Oxaliplatin, and a Fluoropyrimidine (19).
4. A Phase 3, Randomized, Double-Blinded Study of IMC-1121B and Best Supportive Care (BSC) Vs. Placebo and BSC in the Treatment of Metastatic Gastric or Gastroesophageal Junction Adenocarcinoma Following Disease Progression on First Line Platinum- or Fluoropyrimidine-Containing Combination Therapy (20).
5. A Randomized, Multicenter, Double-Blind, Placebo-Controlled Phase 3 Study of Weekly Paclitaxel With or Without Ramucirumab (IMC1121B) Drug Product in Patients With Metastatic Gastric Adenocarcinoma, Refractory to or Progressive After First-Line Therapy With Platinum and Fluoropyrimidine (21).

Demographic, clinical, serological, treatment and survival outcomes were extracted. In each analysis, we excluded patients that had missing information for the biomarkers or outcome being studied.

Each study had already obtained approval from the Institutional Review Board (IRB) of its respective participating centers. Given the retrospective nature of the research, with no intervention and with only de-identified data being provided to the investigators, it was deemed non-human subject research and exempt from IRB review.

### 2.2 Biomarkers and survival outcomes

We studied the baseline neutrophil (N1) and lymphocyte (L1) counts, as well as baseline NLR (NLR1). Baseline indicates the count in the blood test performed just before starting the first dose of the study treatment. We studied the same biomarkers before the second cycle of medication (N2, L2, NLR2; median days after baseline 27 days (21–33)) and before the third cycle (N3, L3, NLR3; median days after baseline 43 days (35–56). We also studied the performance of the percentage of change over NLR1 of the NLR between baseline and the second cycle (PercenNLR1-NLR2) and between baseline and the third cycle (PercenNLR1-NLR3). Biomarkers were studied as continuous and dichotomized as low (< median) or high (≥ median). To prevent differences in baseline biomarker’s levels, we divided patients according to the median within each different studies (1–5) in most of the analysis and according to the median within each cancer type (lung, colorectal or gastric/gastroesophageal junction (GEJ)) in some.

We assessed two survival outcomes: overall survival (OS) and PFS.

### 2.3 Statistical analysis

Categorical variables were described through absolute frequency (relative frequency) while continuous variables were described through median (interquartile range). We selected covariates according to previously reported factors that were available on our database that could influence the relationship of our biomarkers with prognosis in solid tumors: age, sex (male *vs*. female), age at inclusion in study (< 60 *vs*. ≥ 60 years, cut-off of age selected according to previous literature (22, 23)), race (white, black or other) and disease stage according to the TNM staging system. We also stratified patients according to the following study-specific variables that were part of the stratification analysis of each study:

1. *Eastern Cooperative Oncology Group Performance Status Scale* (ECOG PS) just prior to randomization (0 *vs*. 1) and tumor response to induction chemotherapy (complete response/partial response *vs* stable disease).
2. ECOG PS (0 *vs*. 1), gender, prior maintenance therapy (yes *vs*. no), and geographic region (Japan/East Asia *vs*. rest of the world (ROW)).
3. Geographic region (North America *vs*. Europe *vs*. ROW), KRAS status (mutant *vs*. wild-type), and time to disease progression after beginning first-line treatment (< 6 months *vs*. ≥ 6 months).
4. Weight loss (≥ 10% over the prior 3 months *vs*. < 10%), geographic region (North America, Europe, Australia, and New Zealand *vs*. South and Central America, India, Egypt, South Africa, Lebanon, Jordan, and Saudi Arabia *vs*. Asia), and location of the primary tumor (gastric [including tumors of the gastric cardia that extend into the GEJ] *vs*. GEJ [including tumors of the distal esophagus that extend into the GEJ, and tumors involving the GEJ when precise identification of the organ of origin is not possible]).
5. Disease measurability (measurable *vs*. non-measurable disease), geographic region (Europe [including Israel]/North America/Australia *vs*. Asia [including East Asia (Japan, South Korea, China, Hong Kong, Taiwan) and Southeast Asia (Malaysia, Thailand, Singapore] *vs*. ROW [including South America]), and time to progression on first-line therapy (< 6 months *vs*. ≥ 6 months).

All statistical analyses were two-sided, with statistical significance defined as a *p*-value < 0.05. Analyses were conducted using R version 4.4.2.

#### 2.3.1 Kaplan-Meier analysis

Kaplan-Meier (KM) survival curves were constructed to estimate and visualize survival probabilities for different subgroups, stratified by low/high biomarker levels and other clinical characteristics. The KM method accommodates censored data, ensuring unbiased survival estimates despite varying follow-up durations among patients. Ninety-five percent confidence intervals (95 CI%) were included to quantify uncertainty around the survival estimates. Risk tables displayed below each KM plot show the number of patients at risk at regular time intervals for each biomarker group.

Survival differences between groups were assessed using the log-rank test for the entire cohort, along with pairwise comparisons between strata. To summarize and enhance the visualization of pairwise comparisons when more than two strata were present, heatmaps were generated. *p*-values were categorized into predefined ranges (< 0.001, 0.001–0.01, 0.01–0.05, 0.05–0.1, 0.1–0.5, >0.5) and represented by gradient shades of blue. Cells labeled ‘NA’ indicated comparisons where statistical testing was not applicable due to insufficient data.

#### 2.3.2 Cox proportional hazards analysis

Both univariate and multivariate Cox proportional hazards models were used to estimate adjusted hazard ratios (HRs) and their 95% CI for the association between survival outcomes and biomarkers, as well as other cohort characteristics. Potential confounders, as previously described, were first evaluated in univariate analysis. Only variables that were statistically significant in the univariate analysis were included in the multivariate model.

The proportional hazards assumption was assessed using Schoenfeld residuals and their corresponding p-values. Additionally, to explore whether the association between biomarkers and survival outcomes varied across subgroups, the sample was stratified by demographic and clinical characteristics to identify strata where biomarkers had stronger or more significant associations with survival.

#### 2.3.3 Classification performance

We evaluated the ability of each baseline biomarker to predict survival outcomes using receiver operating characteristic (ROC) curves, calculating the area under the curve (AUC) for each biomarker. The biomarker with the highest AUC was identified as the best-performing predictor. DeLong’s test was used to statistically compare AUC values between the top-performing biomarker and the others, with p-values summarizing the results.

The cut-off values for each biomarker were calculated using three distinct approaches to evaluate their performance. The first method was the median cut-off, where the threshold was determined as the median of the biomarker’s distribution following the methodology of previous studies. This method ensures that half of the patients fall above and half below the cut-off, providing a simple and non-parametric approach suitable for baseline comparisons. The second method utilized Youden’s Index, which was calculated from the ROC curve. This cut-off value maximizes Youden’s Index, defined as sensitivity plus specificity minus one, identifying the point on the ROC curve that optimally balances sensitivity and specificity, thereby enhancing overall diagnostic accuracy. The third method involved calculating a balanced cut-off using the “closest-to-top-left” method on the ROC curve. This approach minimizes the Euclidean distance to the top-left corner of the graph, representing perfect sensitivity and specificity, ensuring a balanced trade-off between these two metrics.

To identify the best cut-off for each biomarker, we focused on the metrics most relevant to our study objectives. Sensitivity was given priority due to the high progression and death rate in cancer, as it is crucial to detect as many progression events (true positives) as possible. Positive predictive value (PPV) was also prioritized to ensure that patients identified as high-risk truly had a high likelihood of progression, thereby minimizing unnecessary interventions. While not the sole criterion, the AUC, which reflects the biomarker’s overall ability to distinguish between positive and negative events, was considered when sensitivity and PPV were comparable. The final selection was based on the cut-off value with the highest sensitivity, provided that it maintained acceptable levels of PPV and AUC. In cases where differences in sensitivity were minimal, PPV and AUC served as secondary factors in the decision-making process.

Variables that were statistically associated with survival outcomes in the Cox proportional hazards analysis were used to define population subsets. Subsets were generated based on combinations of these variables (up to three at a time). To ensure statistical robustness, only groups with at least five patients were included in the analysis. The relationship between biomarkers and survival was assessed using logistic regression, and AUC values were calculated for both the entire cohort and each subset. Subsets with an AUC higher than the AUC of the entire cohort were selected for further analysis. To determine whether the AUC of each subset was significantly different from that of the full cohort, permutation testing was performed. For each subset, 1,000 random permutations of the outcome variable were generated, and the observed difference in AUC was compared with the null distribution to compute a *p*-value. Subsets with *p*-values < 0.05 were further examined using ROC curves. For each significant subset, ROC curves comparing the subset with the entire cohort were plotted to visualize differences in classification performance.

## 3 Results

A total of 4,484 patients had data available for the study number distribution: 472 patients from Study 1 (10.30%), 1,253 from Study 2 (27.33%), 1,074 from Study 3 (23.43%), 355 from Study 4 (7.74%), and 1,330 from Study 5 (29.01%). A total of 4,513 patients had data about the cancer type: 1,725 patients had lung cancer (38.22%), 1,088 colorectal cancer (24.11%) and 1,700 Gastric or GEJ cancer (37.69%). Demographics and clinical variables are reflected in **Table 1** divided by study group and cancer type. Some additional demographics and clinical data are detailed in the **Supplementary Table 1**. We included all the factors that were used in each study for performing stratification. Between 3,498 and 3,516 patients were included for the analysis of the baseline biomarkers NLR1, N1 and L1. The exact cut-off values used for dichotomizing the biomarkers as high or low are gathered in **Supplementary Table 2**.

**Table 1:** Demographics and clinical characteristics of the cohort.

Variable	Categories	Study					Cancer type		
		1	2	3	4	5	Lung	Colorectal	Gastric or GEJ
Sex	0	270 (57%)	834 (67%)	617 (57%)	248 (70%)	944 (71%)	1104 (64%)	631 (58%)	1207 (71%)
	1	202 (43%)	419 (33%)	457 (43%)	107 (30%)	386 (29%)	621 (36%)	457 (42%)	493 (29%)
Age		61.34 (55.21-67.94)	62 (55-68)	62.31 (53.87-68.63)	60 (52-69)	61 (53-68)	62 (55-68)	62.31(53.87-68.63)	61 (53-69)
Race	White	449 (95%)	1029 (82%)	817 (76%)	272 (77%)	814 (61%)	1478 (86%)	817 (76%)	1086 (64%)
	Black	3 (1%)	33 (3%)	30 (3%)	6 (2%)	24 (2%)	36 (2%)	30 (3%)	30 (2%)
	Asian	20 (4%)	160 (13%)	214 (20%)	56 (16%)	462 (35%)	180 (10%)	214 (20%)	518 (31%)
	A. Indian or Alaskan	0 (0%)	29 (2%)	1 (0%)	1 (0%)	2 (0%)	29 (2%)	1 (0%)	3 (0%)
	Multiple	0 (0%)	0 (0%)	3 (0%)	0 (0%)	2 (0%)	0 (0%)	3 (0%)	2 (0%)
	Hawaiian or Pacific	0 (0%)	1 (0%)	2 (0%)	0 (0%)	0 (0%)	1 (0%)	2 (0%)	0 (0%)
	Other	0 (0%)	0 (0%)	0 (0%)	20 (6%)	26 (2%)	0 (0%)	0 (0%)	46 (3%)
	Unknown	0 (0%)	1 (0%)	7 (1%)	0 (0%)	0 (0%)	1 (0%)	7 (1%)	0 (0%)
Baseline ECOG PS	0	167 (35%)	1253 (100%)	545 (51%)	98 (28%)	522 (39%)	1420 (82%)	545 (51%)	621 (36%)
	1	305 (65%)	0 (0%)	529 (49%)	256 (72%)	808 (61%)	305 (18%)	529 (49%)	1064 (63%)
Stage	Stage III B	44 (9%)	0 (0%)	0 (0%)	0 (0%)	0 (0%)	44 (3%)	0 (0%)	0 (0%)
	Stage IV	428 (91%)	1253 (100%)	1074 (100%)	355 (100%)	1330 (100%)	1681 (97%)	1074 (100%)	1685 (100%)
Histological subtype	Adenocarcinoma Squamous cell Adenosquamous Poor differentiated/NE Large cell Bronchogenic Epidermoid	405 (86%) - - 27 (5%) 32 (7%) 7 (2%) -	720 (62%) 272 (23%) 17 (2%) 87 (8%) 37 (3%) - 19 (2%)	1014 (95%) - - 59 (5%) - - -	354 (100%) - - - - - -	1330 (100%) - - - - - -	1125 (69%) 272 (17%) 17 (1%) 114 (7%) 69 (4%) 7 (1%) 19 (1%)	1014 (95%) - - 59 (5%) - - -	1684 (100%) - - - - - -
Death event	No	359 (76%)	369 (29%)	303 (28%)	77 (22%)	298 (22%)	713 (41%)	303 (28%)	375 (22%)
	Yes	113 (24%)	884 (71%)	771 (72%)	278 (78%)	1032 (78%)	1012 (59%)	771 (72%)	1310 (78%)
Progression event	No	175 (37%)	112 (9%)	104 (10%)	48 (13%)	180 (14%)	287 (17%)	104 (10%)	228 (14%)
	Yes	297 (63%)	1141 (91%)	970 (90%)	307 (86%)	1150 (86%)	1438 (83%)	970 (90%)	1457 (86%)

The number next to some variables indicates for which study that variable was a stratification factor. Categorical variables are expressed with absolute frequency (relative frequency) and continuous with median (interquartile range). GEJ, Gastroesophageal junction; A. Indian or Alaskan, American Indian or Alaska Native; Hawaiian or Pacific, Native Hawaiian or other Pacific Islanders; NE, not specified; ECOG PS, Eastern Cooperative Oncology Group Performance Status.

### 3.1 Kaplan-Meier analysis

**Figure 1:**
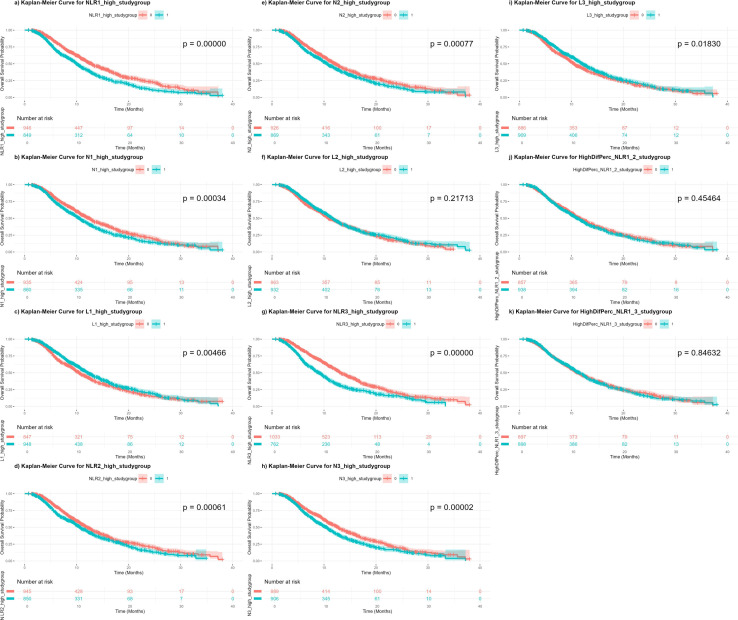
Kaplan-Meier curves of overall survival independent on treatment regimen, according to high or low categories of each biomarker. **(a)** NLR1: baseline neutrophil-to-lymphocyte count; **(b)** N1: baseline neutrophil count; **(c)** L1: baseline lymphocyte count; **(d)** NLR2: neutrophil-to-lymphocyte count at 3 weeks; **(e)** N2: neutrophil count at 3 weeks; **(f)** L2: lymphocyte count at 3 weeks; **(g)** NLR3: neutrophil-to-lymphocyte count at 6 weeks; **(h)** N3: neutrophil count at 6 weeks; **(i)** L3: lymphocyte count at 6 weeks; **(j)** PercenNLR1_NLR2: percentage of the change from NLR1 to NLR2 over the baseline NLR1; **(k)** PercenNLR2_NLR3: percentage of the change from NLR1 to NLR3 over the baseline NLR1.

**Figure 2:**
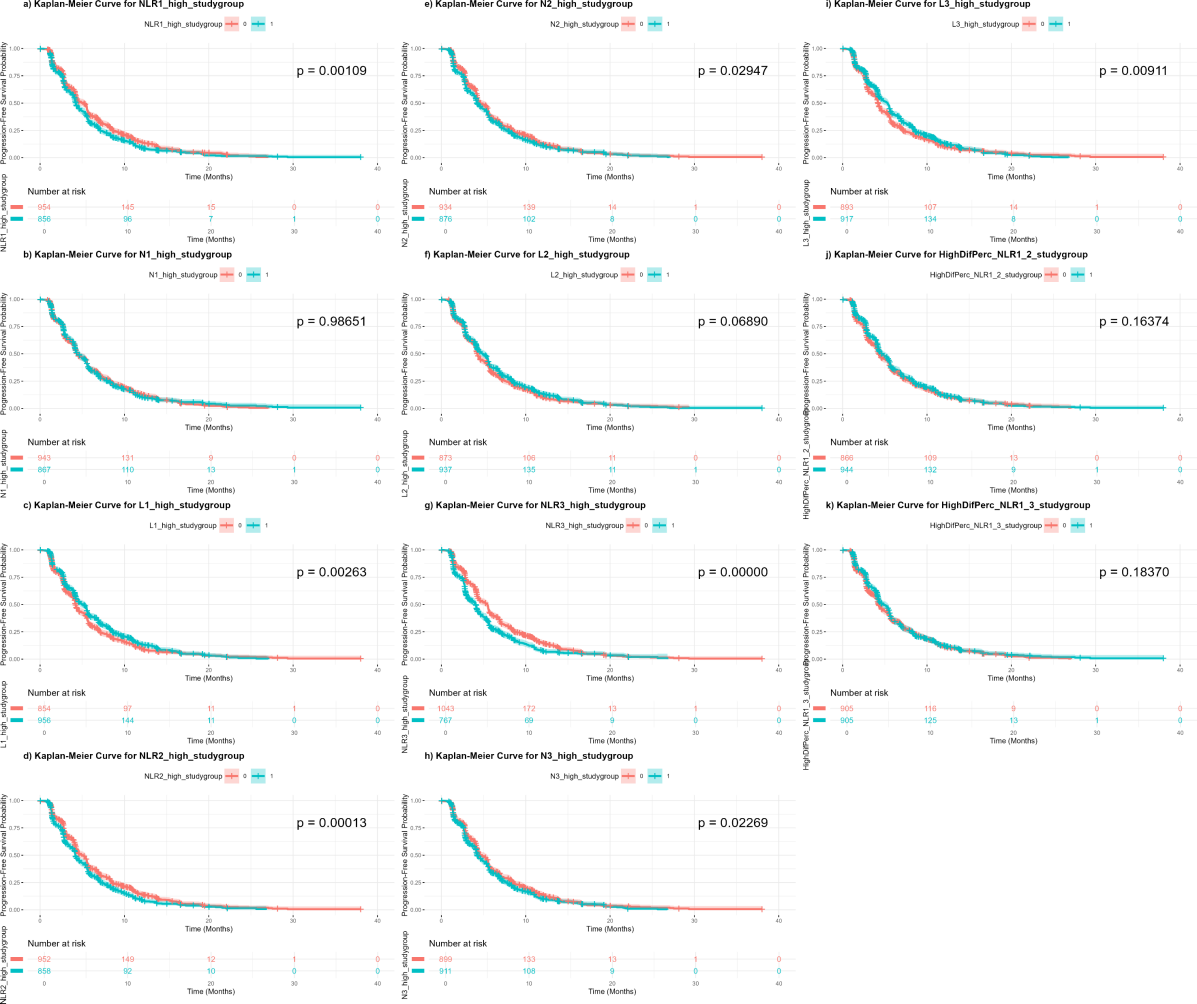
Kaplan-Meier curves of progression-free survival independent on treatment regimen, according to high or low categories of each biomarker. NLR1, baseline neutrophil-to-lymphocyte count; N1, baseline neutrophil count; L1, baseline lymphocyte count; NLR2, neutrophil-to-lymphocyte count at 3 weeks; N2, neutrophil count at 3 weeks; L2, lymphocyte count at 3 weeks; NLR3, neutrophil-to-lymphocyte count at 6 weeks; N3, neutrophil count at 6 weeks; L3, lymphocyte count at 6 weeks; PercenNLR1_NLR2, percentage of the change from NLR1 to NLR2 over the baseline NLR1; PercenNLR2_NLR3, percentage of the change from NLR1 to NLR3 over the baseline NLR1.

For OS, patients in the high categories of neutrophils and NLR and the low category of lymphocytes exhibited significantly worse survival curves, except for L2, which did not reach statistical significance. Among the biomarkers, NLR1 and NLR3 showed the smallest p-values (*p* = < 0.0001). For PFS, similar patterns were observed, though N1 and L2 were not significant, and the smallest p-value was again found for NLR3 (*p* < 0.0001). The percentage-based changes in NLR over different time periods were not statistically significant for either OS or PFS. The KM curves, along with their corresponding *p*-values, are presented in **Figure 1** and **Figure 2**. When stratifying the sample by study group and cancer type, significant differences were observed in both strata of OS and in PFS stratified by cancer type, with survival curves differing significantly between most of high and low biomarker categories (NLR1, N1, and L1) in nearly all subgroups. However, some non-significant differences were found in PFS when stratified by study group (**Supplementary Figures 1**-**4**). We also aimed to explore differences based on histological subtype, although the majority of patients had adenocarcinomas (87.3%), with only a small proportion presenting with squamous cell carcinoma or less common subtypes such as epidermoid, large cell, or bronchogenic lung carcinoma. We found that NLR1, N1, and L1 showed statistically significant differences only in the adenocarcinoma subgroup for OS, while NLR1 and L1 were also significant in epidermoid lung carcinoma (See **Supplementary Figures 5**-**7**). For PFS, the results were more variable, with adenocarcinoma showing significant differences for all three biomarkers, while findings in other histological subtypes were inconsistent (See **Supplementary Figures 8**-**10**).

**Figure 3:**
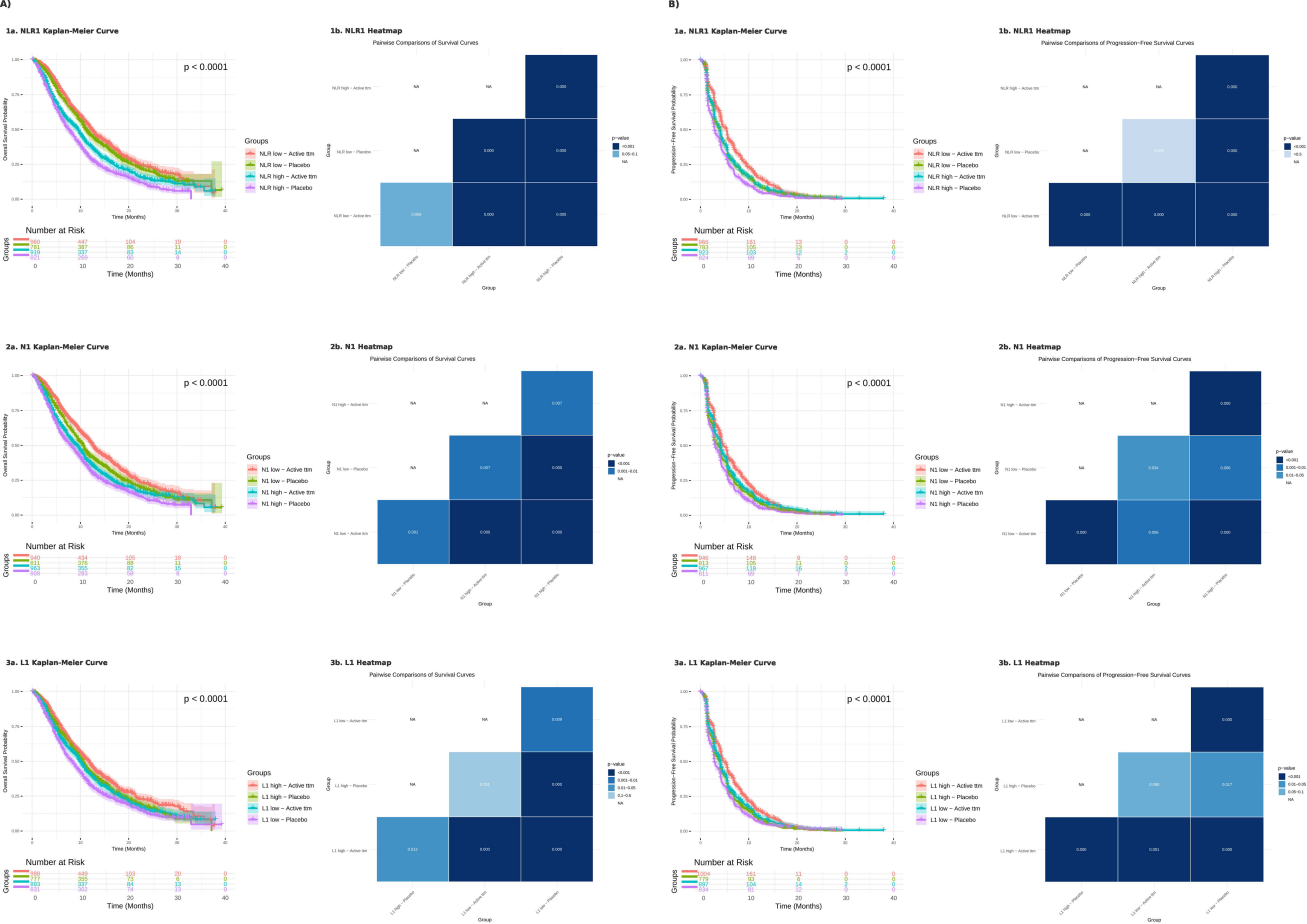
Kaplan-Meier curves of the combination of the two variables treatment (active or placebo) and high/low biomarkers with pairwise comparison among the curves reflected on a heatmap. **(A)** Overall survival; **(B)** Progression-free survival. 1a and 1b) Baseline neutrophil-to-lymphocyte count; 2a and 2b) Baseline neutrophil count; 3a and 3b) Baseline lymphocyte count.

To further analyze these relationships, the cohort was divided into four groups based on the combination of biomarker category (high/low) and treatment status (active treatment/placebo). For OS, survival generally declined following a gradient from biomarker Low – Active treatment, to biomarker Low – Placebo, to biomarker High – Active treatment, to biomarker High – Placebo (reversed for L1). However, some exceptions were noted. For NLR1, no significant difference was observed between the two low NLR1 groups, regardless of treatment status (*p* = 0.059). Similarly, for L1, no significant difference was observed between L1 High – Placebo and L1 Low – Active treatment (*p* = 0.291). In PFS, comparable patterns were seen, with NLR1 and L1 showing no significant differences between the same two groups (*p* = 0.688 and *p* = 0.090, respectively), while all other groups displayed statistically significant differences (**Figure 3**).

### 3.2 Cox proportional hazards analysis

In the Cox proportional hazards analysis, NLR1 was significantly associated with an increased risk of death in both the univariate and multivariate models for OS. As a continuous variable, NLR1 showed a HR of 1.031 (95% CI: 1.024 – 1.038, *p* < 0.001) in the univariate analysis and HR 1.035 (95% CI: 1.028 – 1.042, < 0.001) in the multivariate analysis. In contrast, N1 and L1 did not show significant associations with OS when analyzed as continuous variables. **Supplementary Table 3** presents other variables associated with OS. When analyzed as categorical variables, however, all three biomarkers (NLR1, N1, and L1) were significantly associated with OS. The corresponding HRs in the univariate analysis were 1.508 (95% CI: 1.390 – 1.636, *p* < 0.001) for NLR1, 1.390 (95% CI: 1.282 – 1.507, *p* < 0.001) for N1, and 0.801 (95% CI: 0.739 – 0.868, *p* < 0.001) for L1, with similar values observed in the multivariate analysis (**Supplementary Table 4**).

For PFS, both NLR1 and N1 were significantly associated with PFS when considered as continuous variables, with HR 1.022 (95% CI: 1.016 – 1.029, *p* < 0.001) for NLR1 and HR 1.001 (95% CI: 1.000 – 1.001, *p* = 0.003) for N1 (**Supplementary Table 5**). When analyzed as categorical variables, the HRs were 1.261 (95% CI: 1.174 – 1.355, *p* < 0.001) for NLR1, 1.154 (95% CI: 1.074 – 1.239, *p* < 0.001) for N1, and 0.848 (95% CI: 0.789 – 0.911, *p* < 0.001) for L1 (**Supplementary Table 6**).

Additionally, a Cox proportional hazards analysis incorporating variables that were significant in the univariate analysis revealed that biomarkers were associated with higher HRs of death in specific patient subgroups. However, these high-risk subgroups varied depending on the biomarker, although some common characteristics were being under 60 years old, not White, having Stage IV disease and ECOG PS 1 (**Supplementary Table 7**).

### 3.3 Classification performance

**Figure 4:**
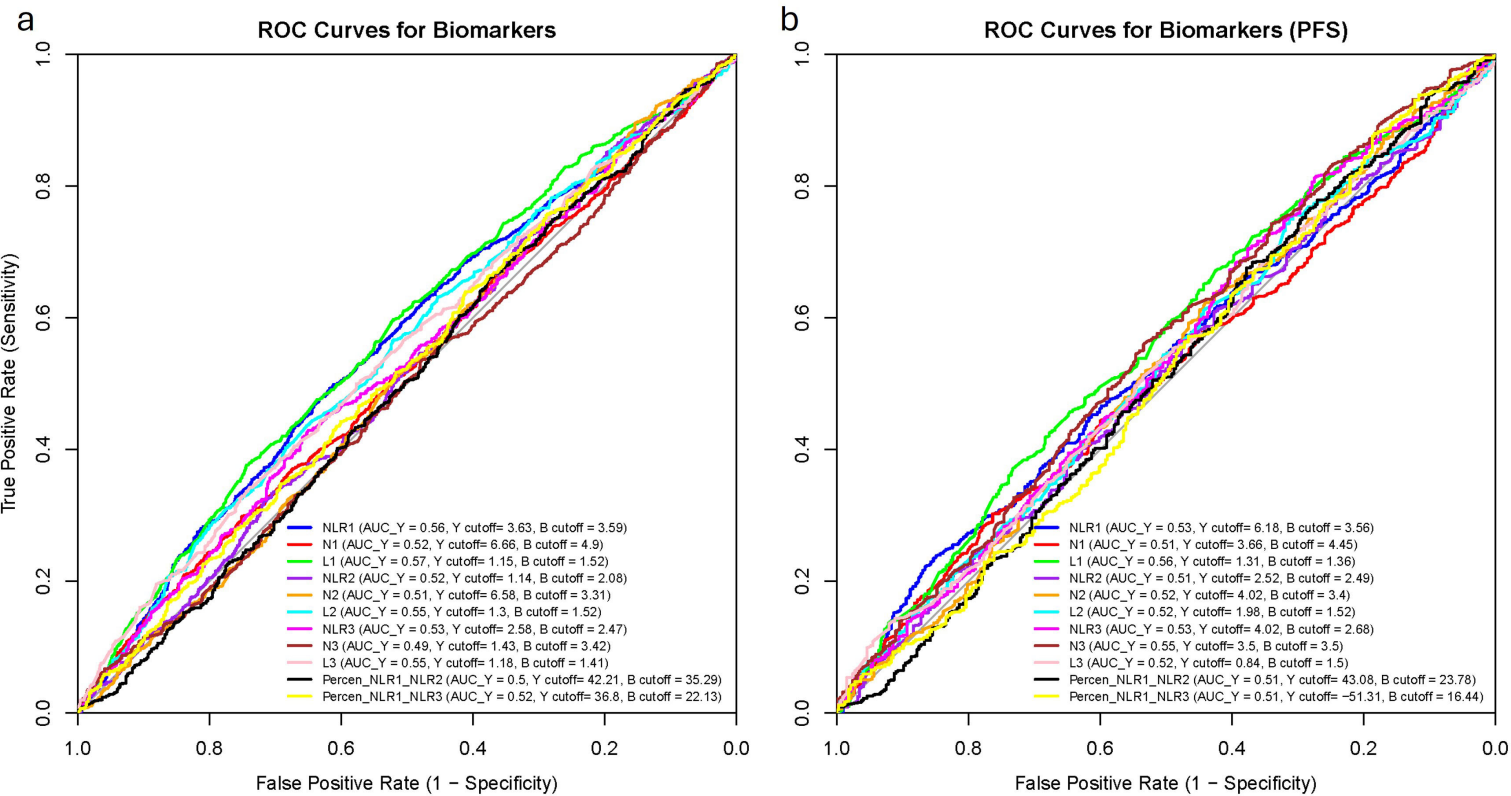
Receiving operating characteristics (ROC) curves of each of the biomarkers with their areas under the curve (AUC) and their cut-off values. **(a)** Overall survival; **(b)** Progression-free survival. AUC_Y, AUC with Youden’s index cut-off value; Y cutoff, Youden’s index cut-off; B cutoff, balanced cut-off; N1, baseline neutrophil count; L1, baseline lymphocyte count, NLR1, baseline neutrophil-to-lymphocyte count; N2, neutrophil count at 3 weeks; L2, lymphocyte count at 3 weeks; NLR2, neutrophil-to-lymphocyte count at 3 weeks; N3, neutrophil count at 6 weeks; L3, lymphocyte count at 6 weeks; NLR3, neutrophil-to-lymphocyte count at 6 weeks; PercenNLR1_NLR2, percentage of the change from NLR1 to NLR2 over the baseline NLR1; PercenNLR2_NLR3, percentage of the change from NLR1 to NLR3 over the baseline NLR1.

**Figure 4** presents the ROC curves for all biomarkers in relation to OS (a) and PFS (b). For OS, AUC values ranged from 0.49 (N3) to 0.57 (L1), with L1 demonstrating statistically significant differences in AUC compared to all biomarkers except for L2, NLR1, and NLR3. In the case of PFS, AUC values varied between 0.51 (N1, NLR2, Percentage NLR1–NLR2, and Percentage NLR1–NLR3) and 0.56 (L1), where L1’s AUC was significantly higher only than those of N1 and NLR1. The *p*-values for the comparison between the biomarkers with the highest AUC and the rest are provided in the **Supplementary Table 8**.

#### 3.3.1 Cut-off values

The optimal cut-off values for NLR1, N1 and L1 were calculated through 3 different methods showing high variability even within the same biomarker for each survival outcome (See **Supplementary Table 9**). The Median and Balanced cut-off values were similar across all biomarkers and survival outcomes, with less than 15% variation between them for most biomarkers. However, some cut-off values derived from the Youden index differed significantly from those calculated by the other two methods. An extreme example was NLR1 for PFS (Youden: 6.13, Median: 3.33, Balanced: 3.56).

When selecting the optimal cut-off based on our criteria—maximizing sensitivity, true predictive value, and AUC—the Median-derived cut-off most frequently demonstrated the best performance, with a few biomarkers favoring the Youden’s cut off.

#### 3.3.2 Subsets

A total of 621 combinations of two or three variables were identified for OS, while 604 combinations were found for PFS. In certain subsets, an AUC of 1.0 was observed; however, due to limited sample sizes and technical constraints in graphing these cases, the corresponding figures were excluded. Instead, we present the next best-performing biomarker subsets, ensuring both clinical relevance and visual interpretability in the ROC curves. For completeness, the numerical data for the subsets with AUC = 1.0 are provided in the **Supplementary Table 10**.

##### 3.3.2.1 OS NLR1

**Figure 5:**
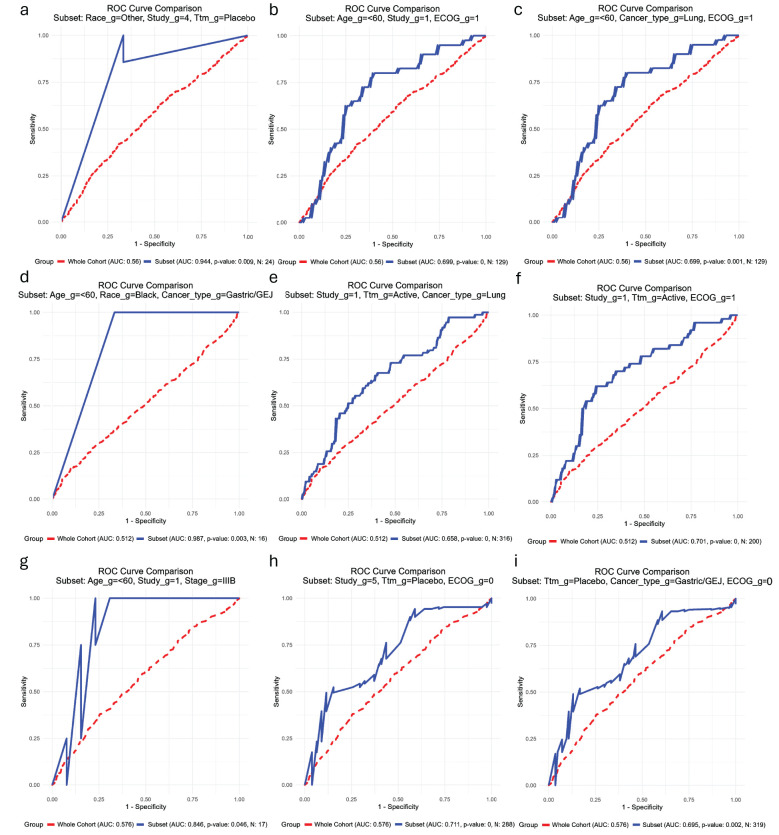
Receiving operating characteristic (ROC) curves showing the subsets of patients (blue) in which each biomarker has the best performance in comparison with their performance in the whole cohort of patients (red) for the outcome overall survival. **(a-c)** Baseline neutrophil-to-lymphocyte ratio; **(d-f)** Baseline lymphocyte count; **(g–i)** Baseline lymphocyte count. _g, group; Ttm, treatment; AUC, area under the curve; ECOG PS, Eastern Cooperative Oncology Group Performance Status; GEJ, gastroesophageal junction.

Fifty-four subsets exhibited significantly higher AUCs compared to the biomarker in the full cohort. The highest AUC was observed in the subset consisting of Other Race (Non-White, Non-Black) + Study 4 + Placebo (AUC = 0.944, n = 24, *p* = 0.009; **Figure 5a**). Among the thirty-four subsets with sample sizes exceeding 100, the highest AUCs were noted in the Under 60 years + Study 1 + ECOG PS 1 group (AUC = 0.6986, n = 129, *p* < 0.001; **Figure 5b**) and the Under 60 years + Lung cancer + ECOG PS 1 group (AUC = 0.6986, n = 129, *p* = 0.001; **Figure 5c**).

##### 3.3.2.2 OS N1

One hundred and sixty-three subsets demonstrated significantly greater AUCs than the biomarker in the general cohort. The highest AUC was identified in the Under 60 years + Black + Gastric/GEJ subgroup (AUC = 0.987, n = 16, *p* = 0.003; **Figure 5d**). Among the one hundred and fifty-three subsets with sample sizes larger than 100, the highest AUCs were observed in the Study Group 1 + Active Treatment + Lung Cancer (AUC = 0.658, n = 316, p < 0.001; **Figure 5e**) and Study Group 1 + Active Treatment + ECOG PS 1 group (AUC = 0.701, n = 200, p < 0.001); *p* < 0.001; **Figure 5f**).

##### 3.3.2.3 OS L1

Only six subsets exhibited significantly higher AUCs than the biomarker in the overall cohort. The highest AUC was observed in the subset consisting of Under 60 years + Study Group 1 + Stage IIIB (AUC = 0.846, n = 17, *p* = 0.0046; **Figure 5g**). Among the three subsets with more than 100 patients, the highest AUCs were found in the Study Group 5 + Placebo + ECOG PS 0 group (AUC = 0.711, n = 288, *p* < 0.001; **Figure 5h**) and the Placebo + Gastric/GEJ Cancer + ECOG PS 0 group (AUC = 0.695, n = 319, *p* = 0.002; **Figure 5i**).

##### 3.3.2.4 PFS NLR1

**Figure 6:**
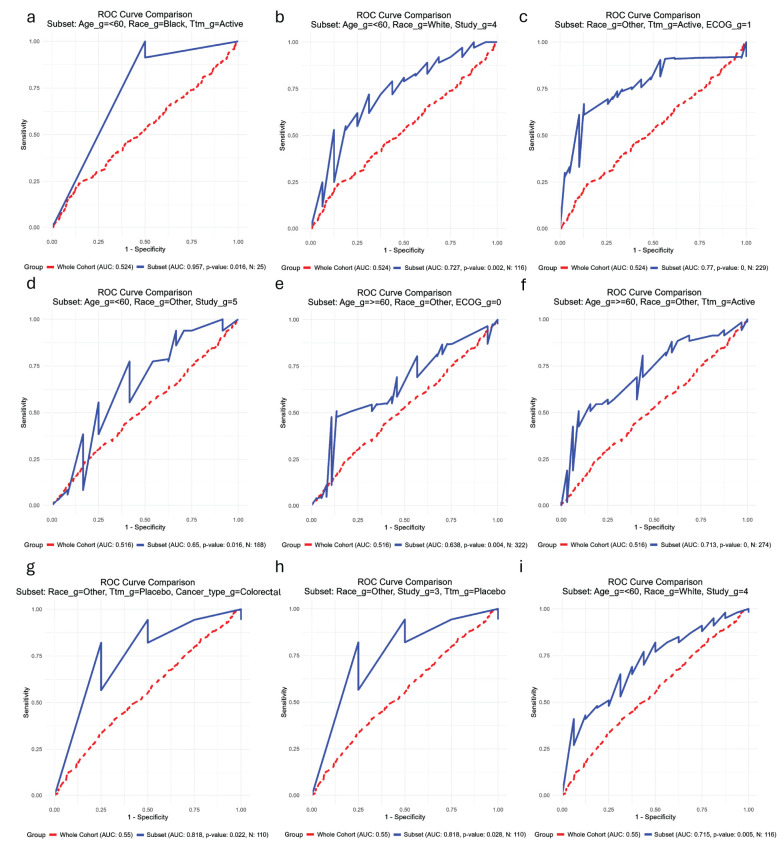
Receiving operating characteristic (ROC) curves showing the subsets of patients (blue) in which each biomarker has the best performance in comparison with their performance in the whole cohort of patients (red) for the outcome progression-free survival. **(a-c)** Baseline neutrophil-to-lymphocyte ratio; **(d-f)** Baseline lymphocyte count; **(g–i)** Baseline lymphocyte count. _g, group; Ttm, treatment; AUC, area under the curve; ECOG PS, Eastern Cooperative Oncology Group Performance Status; GEJ, gastroesophageal junction.

One hundred and forty-three subsets displayed significantly higher AUCs than the biomarker in the general cohort. The highest AUC was observed in the subset Under 60 years + Black + Active Treatment (AUC = 0.957, n = 25, *p* = 0.016; **Figure 6a**). Of the one hundred and thirty-four subsets with sample sizes greater than 100, the highest AUCs were identified in the Other Race (Non-White, Non-Black) + Active Treatment + ECOG PS 1 group (AUC = 0.770, n = 229, *p* < 0.001; **Figure 6b**) and the Under 60 years + White + Study 4 group (AUC = 0.727, n = 116, *p* = 0.002; **Figure 6c**).

##### 3.3.2.5 PFS N1

Eighty-four subsets demonstrated significantly higher AUCs than the biomarker in the full cohort. The highest AUC was observed in the Under 60 years + Other Race (Non-White, Non-Black) + ECOG PS 0 subset (AUC = 0.845, n = 227, *p* < 0.001; **Figure 6d**). Among the seventy-seven subsets with sample sizes above 100, the highest AUCs were observed in the Under 60 years + Other Race (Non-White, Non-Black) + Study 5 group (AUC = 0.766, n = 304, *p* < 0.001; **Figure 6e**) and the Under 60 years + Other Race (Non-White, Non-Black) + Active Treatment group (AUC = 0.713, n = 333, *p* < 0.001; **Figure 6f**).

##### 3.3.2.6 PFS L1

Six subsets exhibited significantly higher AUCs than the biomarker in the general cohort. The highest AUC was identified in the subset consisting of Other Race (Non-White, Non-Black) + Placebo + Colorectal Cancer (AUC = 0.818, n = 110, *p* = 0.003; **Figure 6g**). Among the thirteen subsets with sample sizes exceeding 100, the second and third highest AUCs were found in the Other Race (Non-White, Non-Black) + Study 3 + Placebo group (AUC = 0.818, n = 110, *p* = 0.003; **Figure 6h**) and the Under 60 years + White + Study 4 group (AUC = 0.715, n = 116, *p* = 0.005; **Figure 6i**).

## 4 Discussion

Our findings suggest that biomarkers play a significant role in predicting survival outcomes. While some biomarkers consistently demonstrated significant differences, others, such as L2 in OS and N1/L2 in PFS, did not reach significance, suggesting weaker or context-dependent prognostic value. Additionally, percentage-based changes in NLR over time did not predict survival, suggesting that baseline biomarker levels may be more informative than their longitudinal variations in this cohort. This aligns with previous meta-analyses, which demonstrated a strong prognostic value for NLR1 in multiple cancers, including urothelial carcinoma (muscle invasiveness, OS, recurrence-free survival (RFS) and PFS) (24), endometrial cancer (OS and PFS) (25), head and neck squamous cell carcinoma (OS, disease-free survival (DFS), PFS and cancer-specific survival (CSS)) (26), non-muscle-invasive bladder cancer (RFS and PFS) (27), prostate cancer (OS and RFS) (28) and soft tissue sarcoma (OS and DFS and disease-specific survival (DSS)) (29). However, no meta-analyses have assessed absolute lymphocyte or neutrophil counts, with existing data limited to individual studies analyzing single biomarkers. Howard et al. studied baseline differences in N1 and L1 together with NLR1 according to different patient and disease characteristics, but they only analyzed the association with survival outcomes for the variable NLR1 (15).

Stratified analyses confirmed the predictive value of baseline NLR1, N1, and L1, with significant differences in OS and PFS across most subgroups. These results parallel findings from Howard et al., which showed universally worse OS in patients with above-median NLR across cancer types and other subgroups (15). However, in our study, certain PFS subgroups did not show significance, suggesting that biomarker prognostic performance may vary based on treatment protocols or study-specific factors. Adenocarcinoma was the predominant histological subtype in our sample and consistently demonstrated significant differences in both outcomes between high and low biomarker groups. However, further studies are needed to evaluate these associations in the less represented histological subtypes.

When categorizing patients by biomarker level and treatment status, a clear trend emerged: survival worsened progressively from Low – Active Treatment to High – Placebo, supporting the hypothesis that biomarker category had a greater impact on survival than treatment status itself. This suggests that the effect of treatment is more apparent within the same biomarker category rather than across different biomarker levels. However, treatment still provides a survival advantage within biomarker-defined groups. For NLR1 (OS), survival was similar between Low – Active Treatment and Low – Placebo, suggesting that low NLR1 may independently predict better survival, regardless of treatment. Similarly, for L1 in OS and NLR1 and L1 in PFS, no difference was observed between L1 High or NLR1/N1 Low – Placebo and L1 Low or NLR1/N1 High – Active Treatment, implying that the cumulative number of “high-risk” factors may be more relevant than a single variable. These findings reinforce the notion that biomarker-based risk stratification could be more relevant than treatment alone in certain contexts. Therefore, initiating treatment should be carefully considered as some subgroups show no survival benefit and may face a high risk of side effects.

Cox proportional hazards analysis confirmed NLR1 as an independent predictor of OS, with significant associations in both univariate and multivariate models. While N1 and L1 did not show associations as continuous variables with OS, their categorical analysis revealed significant effects, suggesting a non-linear prognostic value that may be better captured through distinct cutoffs. A similar trend was observed for PFS, emphasizing the importance of clinically meaningful threshold definitions, through finding the best cut-off values.

Biomarker classification performance analysis showed that L1 had the highest AUC for OS and PFS, although differences between biomarkers were mostly not statistically significant, indicating comparable predictive performance for the survival outcomes. The selection of optimal cut-off values revealed that the median cut-off provided a neutral baseline but sometimes was less optimal for clinical decision-making due to its lack of emphasis on either sensitivity or specificity, while Youden’s Index offered a balance between sensitivity and specificity, although sometimes at the expense of sensitivity. The balanced cut-off provided an equal trade-off, ensuring neither metric was disproportionately emphasized. Given the importance of missing a “high-risk” case in cancer, we prioritized sensitivity, PPV, and AUC as the most important metrics. By systematically comparing the selected metrics by the three approaches, we identified the most clinically useful cut-off values for each biomarker. However, these cut-off values varied considerably between biomarkers and the approach, being for NLR1 OS 3.33, 3.63 and 3.59 (Median, Youden and Balanced, respectively), while 3.33, 6.18 and 3.56 for NLR1 PFS. These values align with prior meta-analyses, which reported NLR cut-offs above 3.0 (IQR 2.5–5.0) as valid prognostic indices across solid tumors (12).

In this study, we systematically explored the predictive performance of various biomarkers across multiple patient subgroups using a data-driven approach. Our findings demonstrate that certain demographic and clinical characteristics significantly influence the association between biomarkers and survival outcomes, leading to notable improvements in AUC values in specific subsets. Subgroup analysis of classification performance identified subsets with exceptionally high AUCs, suggesting strong biomarker discrimination in specific populations. Across all biomarkers, age, ECOG PS, race, treatment arm, and cancer subtype emerged as key factors influencing predictive performance. However, given small sample sizes, we prioritized larger subsets (≥ 100 patients) to ensure statistical robustness. The best-performing subsets included a variety of combinations of characteristics for each biomarker and survival outcome, reinforcing the influence of demographic and clinical factors on biomarker predictive value. Prior studies, such as Howard et al., similarly identified higher AUCs almost consistently in Non-White, females, stage III o IV and/or melanoma/pancreatic cancer patients and that they increased further with combinations of “high-risk” demographic or clinical factors, further supporting the role of biomarker stratification in risk assessment (15). Some of these characteristics were not tested in our cohort due to the characteristic not being associated with survival outcomes (sex) or differences in cohort characteristics (types of cancer).

Clinically, these findings suggest that NLR1, N1, and L1 could serve as valuable prognostic biomarkers, particularly when stratified by patient demographics and treatment status. Their variability across subgroups highlights the need for personalized biomarker interpretation in treatment decision-making. Future research should focus on validating optimal cutoffs, assessing their role in treatment selection, and exploring biological mechanisms underlying these associations. Large-scale, prospective studies will be crucial for fully understanding the role of these biomarkers in routine oncology risk assessment.

Our study’s strengths include a comprehensive evaluation of three blood cell components at multiple time points, incorporating dynamic NLR changes rather than analyzing single components in isolation. We also applied three distinct cut-off methods, each emphasizing different performance metrics. Additionally, traditional survival models often assume homogeneous biomarker performance across populations, which may obscure meaningful subgroup-specific associations. By leveraging data-driven stratification, we identified clinically relevant subpopulations where biomarkers demonstrate enhanced predictive accuracy.

However, several limitations should be acknowledged. First, the retrospective nature of the study may introduce unmeasured confounders. Second, while subgroup analysis improved biomarker interpretation and generalizability, some findings may be influenced by small sample sizes, requiring validation in independent cohorts to confirm the clinical utility of these biomarker-defined subgroups. Third, although AUC was used as a primary performance metric, additional measures could enhance the clinical assessment of biomarker utility. Fourth, this study focused on absolute cell counts rather than functional aspects of the immune response, such as neutrophil activation or lymphocyte subtypes. This narrow scope limits mechanistic insight into the biological processes driving prognosis. While absolute cell numbers provide important prognostic information, they do not capture the activation status, functional heterogeneity, or interactions of immune cells, which may be equally or more relevant to clinical outcomes. Finally, the NLR1, N1, and L1 values are highly dynamic and can be influenced by intercurrent conditions such as infections or bone marrow involvement by the tumor, potentially affecting their reliability as prognostic markers. The absence of clinical data to differentiate these scenarios is a limitation and could have introduced variability in biomarker interpretation. Future studies should integrate functional assays and immunophenotyping to provide a more comprehensive and mechanistically informative assessment of biomarker utility.

## 5 Conclusion

These results support the integration of NLR1, N1, and L1 into oncology risk assessment models. However, given the variability in optimal cutoffs and the limitations of cell counts alone, further research should focus on functional immune profiling, prospective validation of cut-off values, and biomarker-driven treatment algorithms. Additionally, the interplay between demographic, clinical, and biomarker characteristics significantly influences predictive accuracy, with some subgroups exhibiting marked improvements in biomarker performance. This highlights the importance of tailoring biomarker interpretation to individual patient characteristics rather than relying on uniform thresholds for risk stratification and treatment decisions.

## Data Availability

The raw data supporting the conclusions of this article will be made available by the authors, without undue reservation.

## Supplementary material

Supplementary Table 1 Demographics and clinical characteristics of the cohort. The number next to some variables indicates for which study that variable was a stratification factor. Categorical variables are expressed with absolute frequency (relative frequency) and continuous with median (interquartile range). GEJ: Gastroesophageal junction; Country^2^ includes East Asia includes Taiwan & Corea. Country^4^: **(a)** North America, Europe, Australia, and New Zealand; **(b)** South and Central America, India, Egypt, South Africa, Lebanon, Jordan, and Saudi Arabia; **(c)** Asia; NA, North America; NLR1, baseline neutrophil-to-lymphocyte count; N1, baseline neutrophil count; L1, baseline lymphocyte count; NLR2, neutrophil-to-lymphocyte count at 3 weeks; N2, neutrophil count at 3 weeks; L2, lymphocyte count at 3 weeks; NLR3, neutrophil-to-lymphocyte count at 6 weeks; N3, neutrophil count at 6 weeks; L3, lymphocyte count at 6 weeks; PercenNLR1_NLR2, percentage of the change from NLR1 to NLR2 over the baseline NLR1; PercenNLR2_NLR3, percentage of the change from NLR1 to NLR3 over the baseline NLR1.

Supplementary Table 2 Cut-off values of each biomarker on our study depending on both the study group and the cancer type. NLR1, baseline neutrophil-to-lymphocyte count; N1, baseline neutrophil count; L1, baseline lymphocyte count; NLR2, neutrophil-to-lymphocyte count at 3 weeks; N2, neutrophil count at 3 weeks; L2, lymphocyte count at 3 weeks; NLR3, neutrophil-to-lymphocyte count at 6 weeks; N3, neutrophil count at 6 weeks; L3, lymphocyte count at 6 weeks; PercenNLR1_NLR2, percentage of the change from NLR1 to NLR2 over the baseline NLR1; PercenNLR2_NLR3, percentage of the change from NLR1 to NLR3 over the baseline NLR1; NE, not specified.

Supplementary Table 3 Univariate COX hazard analysis of overall survival using baseline neutrophil-to-lymphocyte ratio (NLR1), neutrophil (N1) count and lymphocyte (L1) count as continuous variables. The variables that were significant in the univariate analysis were then tested in the multivariate analysis. Two models were used to test all variables with NLR1 and all variables with N1 and L1 to avoid multicollinearity issues. The multivariate results between both models did not vary significantly but the ones represented here at those with NLR1. Bold means statistically significant results with p < 0.05. HR, hazard ratios; 95% CI HR, 95% confidence intervals of hazard ratios; NA, not available; y, years; ECOG PS, Eastern Cooperative Oncology Group Performance Status; NLR1, baseline lymphocyte-to-neutrophil ratio; N1, baseline neutrophil count; L1, baseline lymphocyte count.

Supplementary Table 4 Univariate COX hazard analysis of overall survival using baseline high neutrophil-to-lymphocyte ratio (NLR1), high neutrophil (N1) count and high lymphocyte (L1) count as categorical variables. The variables that were significant in the univariate analysis were then tested in the multivariate analysis. Two models were used to test all variables with NLR1 and all variables with N1 and L1 to avoid multicollinearity issues. The multivariate results between both models did not vary significantly but the ones represented here at those with NLR1. Bold means statistically significant results with p < 0.05. HR, hazard ratios; 95% CI HR, 95% confidence intervals of hazard ratios; NA, not available.

Supplementary Table 5 Univariate COX hazard analysis of progression-free survival using baseline neutrophil-to-lymphocyte ratio (NLR1), neutrophil (N1) count and lymphocyte (L1) count as continuous variables. The variables that were significant in the univariate analysis were then tested in the multivariate analysis. Two models were used to test all variables with NLR1 and all variables with N1 and L1 to avoid multicollinearity issues. The multivariate results between both models did not vary significantly but the ones represented here at those with NLR1. Bold means statistically significant results with p < 0.05. HR, hazard ratios; 95% CI HR, 95% confidence intervals of hazard ratios; NA, not available.

Supplementary Table 6 Univariate COX hazard analysis of progression-free survival using baseline high neutrophil-to-lymphocyte ratio (NLR1), high neutrophil (N1) count and high lymphocyte (L1) count as categorical variables. The variables that were significant in the univariate analysis were then tested in the multivariate analysis. Two models were used to test all variables with NLR1 and all variables with N1 and L1 to avoid multicollinearity issues. The multivariate results between both models did not vary significantly but the ones represented here at those with NLR1. Bold means statistically significant results with p < 0.05. HR, hazard ratios; 95% CI HR, 95% confidence intervals of hazard ratios; NA, not available.

Supplementary Table 7 Multivariate COX hazard analysis of the variables that were significant in the multivariate analysis divided into subsets. We show the hazard ratios, 95% confidence interval and p-values of the subsets in relation to overall survival of baseline high neutrophil-to-lymphocyte ratio (NLR1), high neutrophil (N1) count and high lymphocyte (L1) count according to the median of each study group except for cancer type where high was categorized according to the median of the cancer type. Bold means statistically significant results with p < 0.05. *p*-val., *p*-value; HR, hazard ratios; 95% CI HR, 95% confidence intervals of hazard ratios; NA, not available. ECOG PS, Eastern Cooperative Oncology Group Performance Status.

Supplementary Table 8
*p*-values of the areas under the curve (AUC) of the biomarkers with the highest AUC (L1) *vs*. the other biomarkers for both overall survival and progression-free survival. Bold indicates *p-values* < 0.05. NLR1, baseline neutrophil-to-lymphocyte count; N1, baseline neutrophil count; L1, baseline lymphocyte count; NLR2, neutrophil-to-lymphocyte count at 3 weeks; N2, neutrophil count at 3 weeks; L2, lymphocyte count at 3 weeks; NLR3, neutrophil-to-lymphocyte count at 6 weeks; N3, neutrophil count at 6 weeks; L3, lymphocyte count at 6 weeks; PercenNLR1_NLR2, percentage of the change from NLR1 to NLR2 over the baseline NLR1; PercenNLR2_NLR3, percentage of the change from NLR1 to NLR3 over the baseline NLR1.

Supplementary Table 9 Optimal cut-off values fo for overall survival and progression-free survival calculated according to three methods: the Median, Youden’s index and a Balanced cut-off. In bold is represented the best cut-off value prioritizing sensitivity, positive predictive value and area under the curve. PPV, positive predictive value; NPV, negative predictive value; AUC, area under the curve; OS, overall survival; PFS, progression-free survival; NLR1, baseline neutrophil-to-lymphocyte count; N1, baseline neutrophil count; L1, baseline lymphocyte count.

Supplementary Table 10 Subsets of patients in which the biomarkers had an area under the curve (AUC) of 1. OS, overall survival; NLR1, baseline neutrophil-to-lymphocyte count; N1, baseline neutrophil count; L1, baseline lymphocyte count; PFS, progression-free survival; ECOG PS, Eastern Cooperative Oncology Group Performance Status.

Supplementary Figure 1 Kaplan-Meier curves of overall survival according to high/low biomarkers and cancer type. NLR1, baseline lymphocyte-to-neutrophil ratio; N1, baseline neutrophil count; L1, baseline lymphocyte count.

Supplementary Figure 2 Kaplan-Meier curves of progression-free survival according to high/low biomarkers and cancer type. Studygroup refers to the median calculated according to the median in each Study group. NLR1, baseline lymphocyte-to-neutrophil ratio; N1, baseline neutrophil count; L1, baseline lymphocyte count.

Supplementary Figure 3 Kaplan-Meier curves of progression-free survival according to high/low biomarkers and study group. NLR1, baseline lymphocyte-to-neutrophil ratio; N1, baseline neutrophil count; L1, baseline lymphocyte count.

Supplementary Figure 4 Kaplan-Meier curves of progression-free survival according to high/low biomarkers and cancer type. Studygroup refers to the median calculated according to the median in each Study group. NLR1, baseline lymphocyte-to-neutrophil ratio; N1, baseline neutrophil count; L1, baseline lymphocyte count.

Supplementary Figure 5 Kaplan-Meier curves of overall survival according to high/low NLR1 (baseline lymphocyte-to-neutrophil ratio) and histological subtype. Studygroup refers to the median calculated according to the median in each Study group.

Supplementary Figure 6 Kaplan-Meier curves of overall survival according to high/low N1 (baseline neutrophil count) and histological subtype. Studygroup refers to the median calculated according to the median in each Study group.

Supplementary Figure 7 Kaplan-Meier curves of overall survival according to high/low L1 (*baseline lymphocyte count)* and histological subtype. Studygroup refers to the median calculated according to the median in each Study group.

Supplementary Figure 8 Kaplan-Meier curves of progression-free survival according to high/low NLR1 (baseline lymphocyte-to-neutrophil ratio) and histological subtype. Studygroup refers to the median calculated according to the median in each Study group.

Supplementary Figure 9 Kaplan-Meier curves of progression-free survival according to high/low N1 baseline neutrophil count) and histological subtype. Studygroup refers to the median calculated according to the median in each Study group.

Supplementary Figure 10 Kaplan-Meier curves of progression-free survival according to high/low L1 baseline lymphocyte count) and histological subtype. Studygroup refers to the median calculated according to the median in each Study group.
