# Supplementary material for: Performance of the neutrophil-to-lymphocyte ratio as a prognostic tool for survival in solid cancers
Source: Front Oncol. 2025 Jul 21;15:1616477. doi: 10.3389/fonc.2025.1616477 (PMC12318771; doi:10.3389/fonc.2025.1616477)
Supplement: Supplementary Table 1 — Demographics and clinical characteristics of the cohort. The number next to some variables indicates for which study that variable was a stratification factor. Categorical variables are expressed with absolute frequency (relative frequency) and continuous with median (interquartile range). GEJ: Gastroesophageal junction; Country2 includes East Asia includes Taiwan & Corea. Country4: (a) North America, Europe, Australia, and New Zealand; (b) South and Central America, India, Egypt, South Africa, Lebanon, Jordan, and Saudi Arabia; (c) Asia; NA, North America; NLR1, baseline neutrophil-to-lymphocyte count; N1, baseline neutrophil count; L1, baseline lymphocyte count; NLR2, neutrophil-to-lymphocyte count at 3 weeks; N2, neutrophil count at 3 weeks; L2, lymphocyte count at 3 weeks; NLR3, neutrophil-to-lymphocyte count at 6 weeks; N3, neutrophil count at 6 weeks; L3, lymphocyte count at 6 weeks; PercenNLR1_NLR2, percentage of the change from NLR1 to NLR2 over the baseline NLR1; PercenNLR2_NLR3, percentage of the change from NLR1 to NLR3 over the baseline NLR1. [file DataSheet1.docx]

Supplementary material

|  |  | Study | | | | | | | Cancer type | | | | | | |
| --- | --- | --- | --- | --- | --- | --- | --- | --- | --- | --- | --- | --- | --- | --- | --- |
|  |  |  | | | | | | |  | | | | | | |
|  |  | 1 | 2 | 3 | 4 | | 5 | | Lung | | Colorectal | | Gastric or GEJ | | |
| Response to induction ^1^ | Stable disease | 243 (53%) |  |  | |  | |  | | 243 (53%) | |  | |  |  |
|  | Partial or complete response | 215 (47%) |  |  | |  | |  | | 215 (47%) | |  | |  |  |
| Country ^2^ | No East Asia |  | 1164 (93%) |  | |  | |  | | 1164 (93%) | |  | |  |  |
|  | East Asia |  | 89 (7%) |  | |  | |  | | 89 (7%) | |  | |  |  |
| Prior maintenance ^2^ | No |  | 975 (78%) |  | |  | |  | | 975 (78%) | |  | |  |  |
|  | Yes |  | 278 (22%) |  | |  | |  | | 278 (22%) | |  | |  |  |
| Country ^3^ | North America |  |  | 285 (27%) | |  | |  | |  | | 285 (44%) | |  |  |
|  | Europe |  |  | 469 (44%) | |  | |  | |  | | 469 (52%) | |  |  |
|  | Rest of world |  |  | 316 (29%) | |  | |  | |  | | 122 (14%) | |  |  |
| Mutant KRAS ^3^ | No mutant wild type |  |  | 541 (51%) | |  | |  | |  | | 541 (51%) | |  |  |
|  | Mutant |  |  | 529 (49%) | |  | |  | |  | | 529 (49%) | |  |  |
| Progression after 1^st^ line therapy ^3^ | < 6 months |  |  | 250 (24%) | |  | |  | |  | | 250 (24%) | |  |  |
|  | ≥ 6 months |  |  | 807 (76%) | |  | |  | |  | | 807 (76%) | |  |  |
| Weight loss ^4^ | < 10% |  |  |  | | 294 (83%) | |  | |  | |  | | 294 (83%) |  |
|  | ≥ 10% prior 3 months |  |  |  | | 61 (17%) | |  | |  | |  | | 61 (17%) |  |
| Country ^4^ | a |  |  |  | | 245 (69%) | |  | |  | |  | | 245 (69%) |  |
|  | b |  |  |  | | 84 (24%) | |  | |  | |  | | 84 (24%) |  |
|  | c |  |  |  | | 26 (7%) | |  | |  | |  | | 26 (7%) |  |
| Location tumor ^4^ | Gastric |  |  |  | |  | | 265 (75%) | |  | |  | | 265 (75%) |  |
|  | GEJ |  |  |  | |  | | 90 (25%) | |  | |  | | 90 (25%) |  |
| Measurable disease ^5^ | No |  |  |  | |  | | 250 (19%) | |  | |  | | 250 (19%) |  |
|  | Yes |  |  |  | |  | | 1080 (81%) | |  | |  | | 1080 (81%) |  |
| Country ^5^ | Europe/NA/Australia |  |  |  | |  | | 796 (60%) | |  | |  | | 796 (60%) |  |
|  | Asia |  |  |  | |  | | 446 (34%) | |  | |  | | 446 (34%) |  |
|  | ROW |  |  |  | |  | | 88 (6%) | |  | |  | | 88 (6%) |  |
| Progression after 1^st^ line therapy ^5^ | < 6 months |  |  |  | |  | | 1012 (76%) | |  | |  | | 1012 (76%) |  |
|  | ≥ 6 months |  |  |  | |  | | 318 (24%) | |  | |  | | 318 (24%) |  |
| Blood components | NLR1 | 3.56 (2.38-5.68) | 4.48 (2.27-8.76) | 2.69 (1.91-4.11) | | 2.93 (1.87-4.61) | | 3.59 (2.16-5.12) | | 4.01 (2.32-7.75) | | 2.69 (1.91-4.11) | | 3.42 (2.07-5.02) |  |
|  | N1 | 6.03 (4.67-7.89) | 5.98 (3.5-9.3) | 4.1 (3.1-5.4) | | 4.4 (3.2-6) | | 4.7 (3.2-6.17) | | 5.99 (3.9-8.81) | | 4.1 (3.1-5.4) | | 4.58 (3.2-6.1) |  |
|  | L1 | 1.64 (1.21-2.15) | 1.24 (0.82-1.8) | 1.5 (1.12-1.93) | | 1.47 (1-1.94) | | 1.35 (1-1.8) | | 1.38 (0.91-1.9) | | 1.5 (1.12-1.93) | | 1.39 (1-1.82) |  |
|  | NLR2 | 2.91 (1.84-5.2) |  | 1.74 (1.16-2.66) | | 2.81 (2.01-4.51) | | 2.47 (1.64-4.15) | | 2.91 (1.84-5.2) | | 1.74 (1.16-2.66) | | 2.63 (1.71-4.33) |  |
|  | N2 | 4.79 (2.91-7.27) |  | 2.46 (1.75-3.5) | | 4.45 (3.36-5.84) | | 3.7 (2.58-4.8) | | 4.79 (2.91-7.27) | | 2.46 (1.75-3.5) | | 3.9 (2.81-5.2) |  |
|  | L2 | 1.51 (1.12-2.07) |  | 1.42 (1.05-1.91) | | 1.51 (1.09-2.02) | | 1.47 (0.98-2) | | 1.51 (1.12-2.07) | | 1.42 (1.05-1.91) | | 1.5 (1-2.01) |  |
|  | NLR3 | 2.39 (1.54-4.09) |  | 1.87 (1.25-2.83) | | 2.6 (1.78-4.29) | | 2.48 (1.67-4.25) | | 2.39 (1.54-4.09) | | 1.87 (1.25-2.83) | | 2.56 (1.71-4.26) |  |
|  | N3 | 3.66 (2.48-5.68) |  | 2.60 (1.91-3.71) | | 4.33 (3.24-5.54) | | 3.83 (2.7-5.2) | | 3.66 (2.48-5.68) | | 2.6 (1.91-3.71) | | 3.99 (2.94-5.4) |  |
|  | L3 | 1.5 (1.1-2) |  | 1.43 (1.09-1.9) | | 1.57 (1.12-2.04) | | 1.47 (1-2.04) | | 1.5 (1.1-2) | | 1.43 (1.09-1.9) | | 1.52 (1.03-2.04) |  |
|  | Percen NLR1_NLR2 | 18.82  (-43.65-54.33) |  | 35.13  (4.23-56.26) | | -3.98 (-41.65-23.20) | | 21.13 (-16.11-47.54) | | 18.82 (-43.65-54.33) | | 35.13 (4.23-56.26) | | 10.71 (-25.32-42.21) |  |
|  | Percen NLR1_NLR3 | 34.51  (-10.50-59.29) |  | 29.32  (-5.19-50.56) | | -2.46 (-35.82-28.85) | | 14.43 (-26.39-44.44) | | 34.51 (-10.50-59.29) | | 29.32 (-5.19-50.56) | | 10.14 (-34.40-37.15) |  |

Supplementary Table 1: Demographics and clinical characteristics of the cohort. The number next to some variables indicates for which study that variable was a stratification factor. Categorical variables are expressed with absolute frequency (relative frequency) and continuous with median (interquartile range). GEJ: Gastroesophageal junction; Country^2^ includes East Asia includes Taiwan & Corea. Country^4^: a) North America, Europe, Australia, and New Zealand; b) South and Central America, India, Egypt, South Africa, Lebanon, Jordan, and Saudi Arabia; c) Asia; NA: North America; NLR1: baseline neutrophil-to-lymphocyte count; N1: baseline neutrophil count; L1: baseline lymphocyte count; NLR2: neutrophil-to-lymphocyte count at 3 weeks; N2: neutrophil count at 3 weeks; L2: lymphocyte count at 3 weeks; NLR3: neutrophil-to-lymphocyte count at 6 weeks; N3: neutrophil count at 6 weeks; L3: lymphocyte count at 6 weeks; PercenNLR1_NLR2: percentage of the change from NLR1 to NLR2 over the baseline NLR1; PercenNLR2_NLR3: percentage of the change from NLR1 to NLR3 over the baseline NLR1.

| Variables | NLR1 | N1 | L1 | NLR2 | N2 | L2 | NLR3 | N3 | L3 | Percen NLR1_NLR2 | Percen NLR1_NLR3 |
| --- | --- | --- | --- | --- | --- | --- | --- | --- | --- | --- | --- |
| Study group |  |  |  |  |  |  |  |  |  |  |  |
| Study Group 1 | 3.56 | 6.03 | 1.64 | 2.91 | 4.79 | 1.51 | 2.39 | 3.66 | 1.5 | 18.82 | 34.51 |
| Study Group 2 | 4.49 | 5.98 | 1.25 | – | – | – | – | – | – | – | – |
| Study Group 3 | 2.69 | 4.1 | 1.5 | 1.75 | 2.46 | 1.42 | 1.87 | 2.6 | 1.43 | 35.13 | 29.32 |
| Study Group 4 | 2.93 | 4.4 | 1.47 | 2.81 | 4.45 | 1.51 | 2.6 | 4.33 | 1.57 | -3.98 | -2.46 |
| Study Group 5 | 3.6 | 4.7 | 1.35 | 2.48 | 3.7 | 1.47 | 2.48 | 3.83 | 1.47 | 21.13 | 14.43 |
| Whole cohort | 3.45 | 5.04 | 1.44 | 2.49 | 3.85 | 1.48 | 2.34 | 3.61 | 1.49 | 17.78 | 18.95 |
| Cancer type |  |  |  |  |  |  |  |  |  |  |  |
| Lung | 4.01 | 5.99 | 1.38 | 2.91 | 4.79 | 1.51 | 2.39 | 3.66 | 1.5 | 18.82 | 34.51 |
| Colorectal | 2.69 | 4.1 | 1.5 | 1.75 | 2.46 | 1.42 | 1.87 | 2.6 | 1.43 | 35.13 | 29.32 |
| Gastric/GEJ | 3.42 | 4.58 | 1.39 | 2.63 | 3.9 | 1.5 | 2.56 | 3.99 | 1.52 | 10.71 | 10.14 |
| Histological type |  |  |  |  |  |  |  |  |  |  |  |
| Adenocarcinoma | 3.22 | 4.70 | 1.46 | 2.19 | 3.31 | 1.46 | 2.19 | 3.24 | 1.49 | 23.71 | 24.49 |
| Squamous cell | 4.97 | 6.62 | 1.13 | – | – | – | – | – | – | – | – |
| Adenosquamous | 5.07 | 5.75 | 1.34 | – | – | – | – | – | – | – | – |
| Poorly differentiated/NE | 3.60 | 5.03 | 1.37 | 2.24 | 2.97 | 1.40 | 2.04 | 3.08 | 1.39 | 30.00 | 34.00 |
| Large cell | 3.33 | 5.87 | 1.60 | 3.72 | 5.47 | 1.34 | 2.81 | 3.50 | 1.51 | 6.61 | 45.12 |
| Bronchogenic | 4.46 | 4.29 | 1.57 | 4.46 | 5.80 | 1.69 | 2.70 | 4.80 | 1.42 | 17.61 | 25.64 |
| Whole cohort | 3.37 | 4.89 | 1.42 | 2.43 | 3.72 | 1.48 | 2.27 | 3.42 | 1.48 | 21.55 | 24.66 |

Supplementary Table 2: Cut-off values of each biomarker on our study depending on both the study group and the cancer type. *NLR1: baseline neutrophil-to-lymphocyte count; N1: baseline neutrophil count; L1: baseline lymphocyte count; NLR2: neutrophil-to-lymphocyte count at 3 weeks; N2: neutrophil count at 3 weeks; L2: lymphocyte count at 3 weeks; NLR3: neutrophil-to-lymphocyte count at 6 weeks; N3: neutrophil count at 6 weeks; L3: lymphocyte count at 6 weeks; PercenNLR1_NLR2: percentage of the change from NLR1 to NLR2 over the baseline NLR1; PercenNLR2_NLR3: percentage of the change from NLR1 to NLR3 over the baseline NLR1; NE: not specified.*


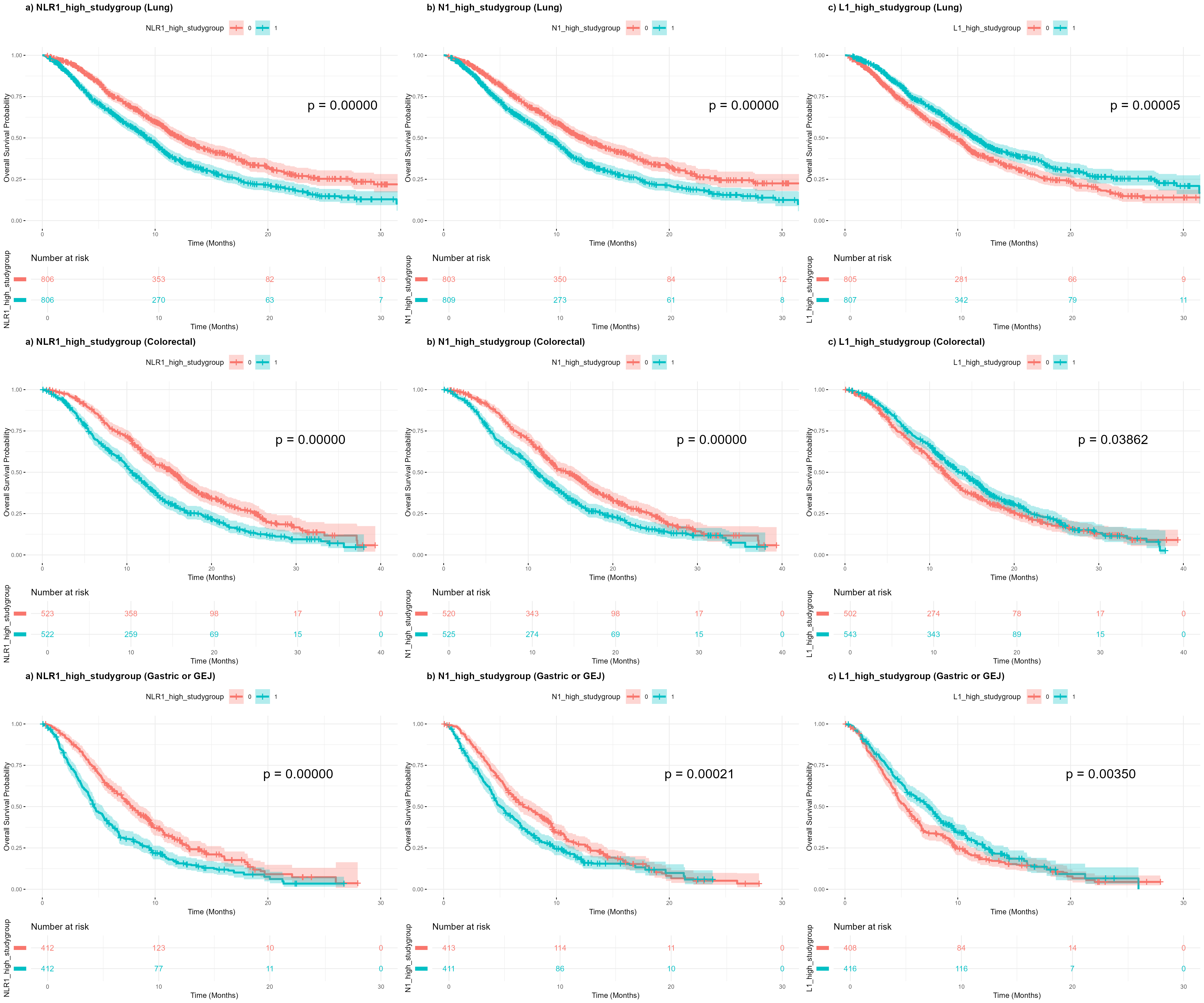

Supplementary Figure 1: Kaplan-Meier curves of overall survival according to high/low biomarkers and cancer type. *NLR1: baseline lymphocyte-to-neutrophil ratio; N1: baseline neutrophil count; L1: baseline lymphocyte count.*


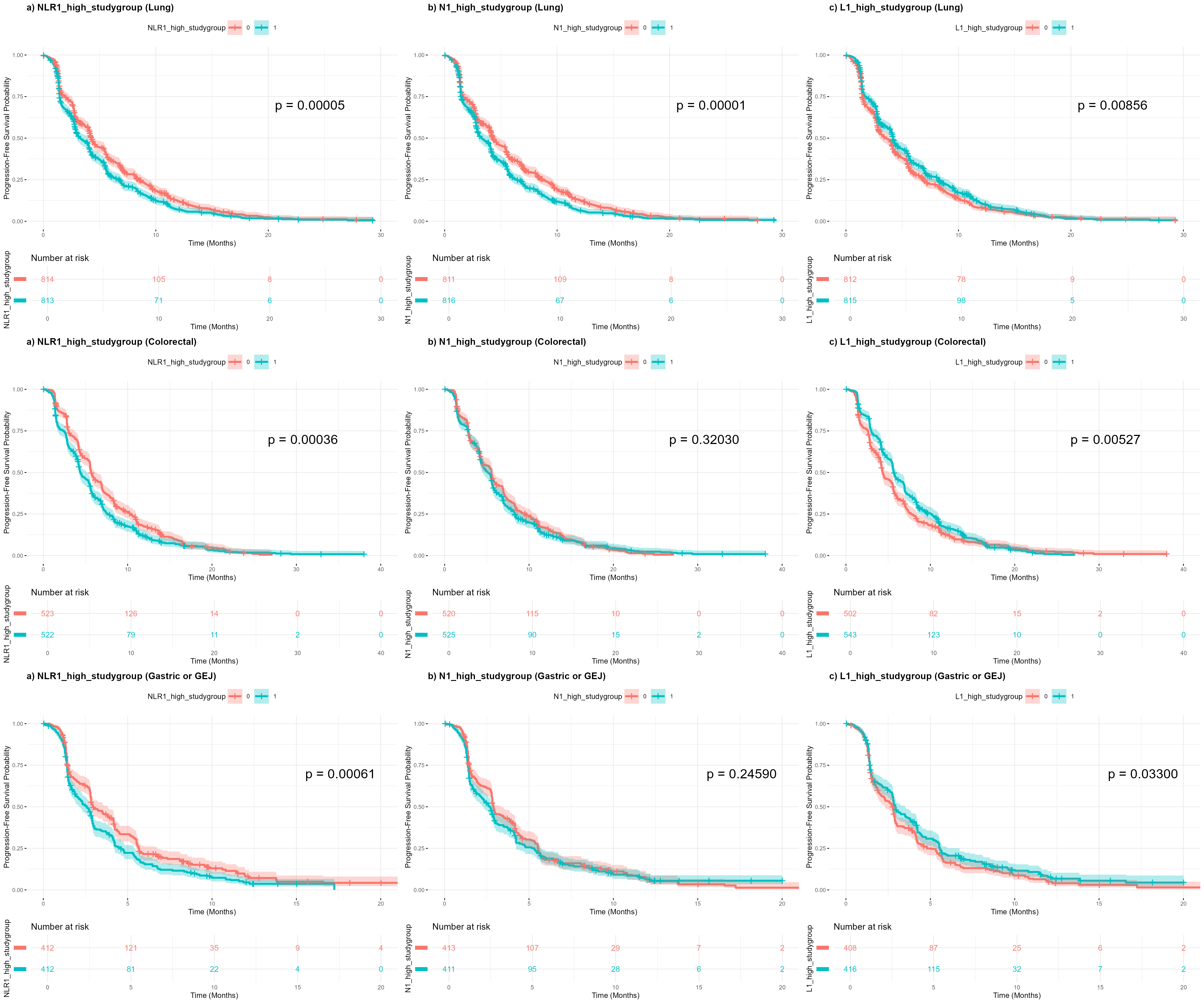

Supplementary Figure 2: Kaplan-Meier curves of progression-free survival according to high/low biomarkers and cancer type. Studygroup refers to the median calculated according to the median in each Study group. *NLR1: baseline lymphocyte-to-neutrophil ratio; N1: baseline neutrophil count; L1: baseline lymphocyte count.*


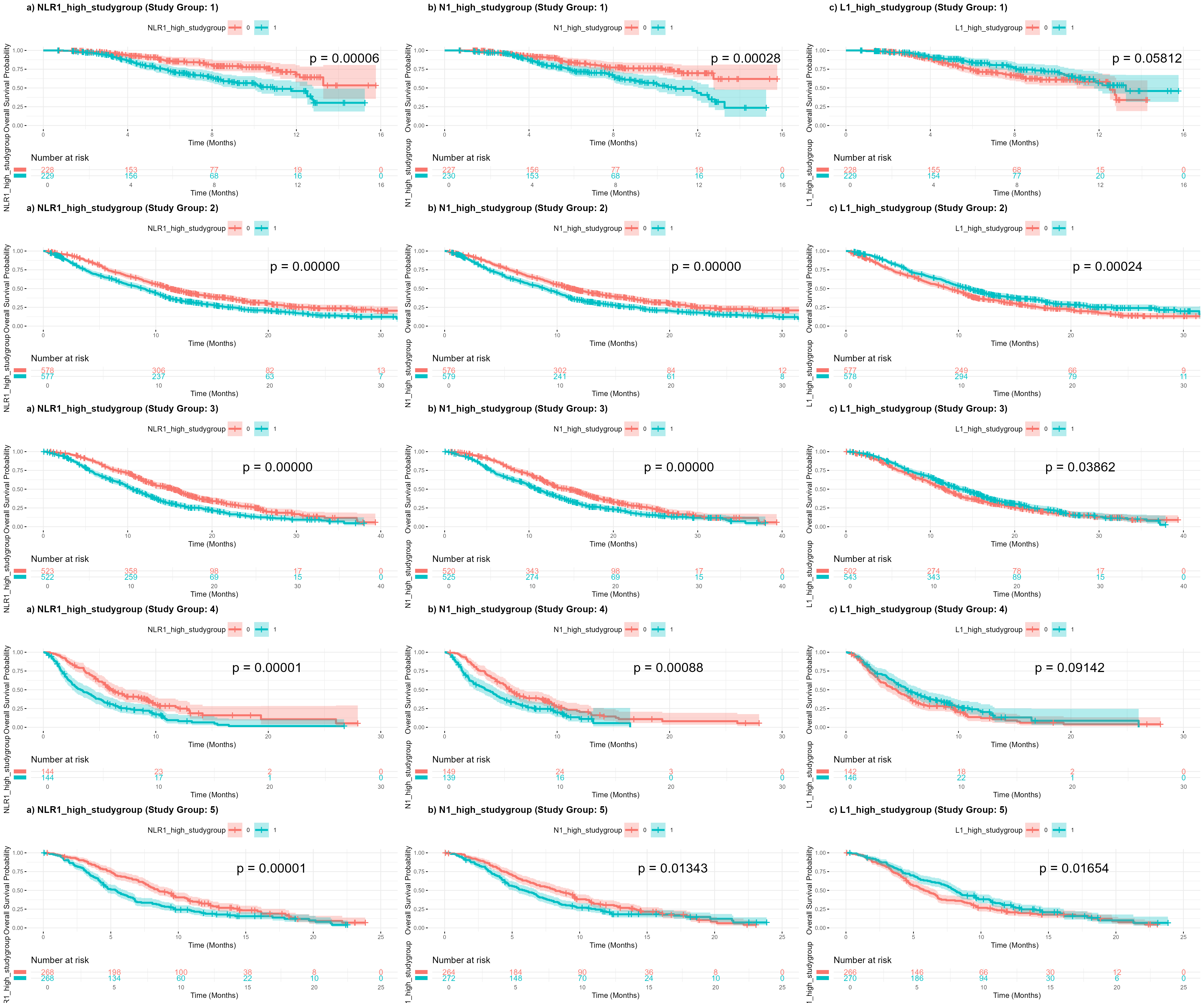


Supplementary Figure 3: Kaplan-Meier curves of progression-free survival according to high/low biomarkers and study group. *NLR1: baseline lymphocyte-to-neutrophil ratio; N1: baseline neutrophil count; L1: baseline lymphocyte count.*


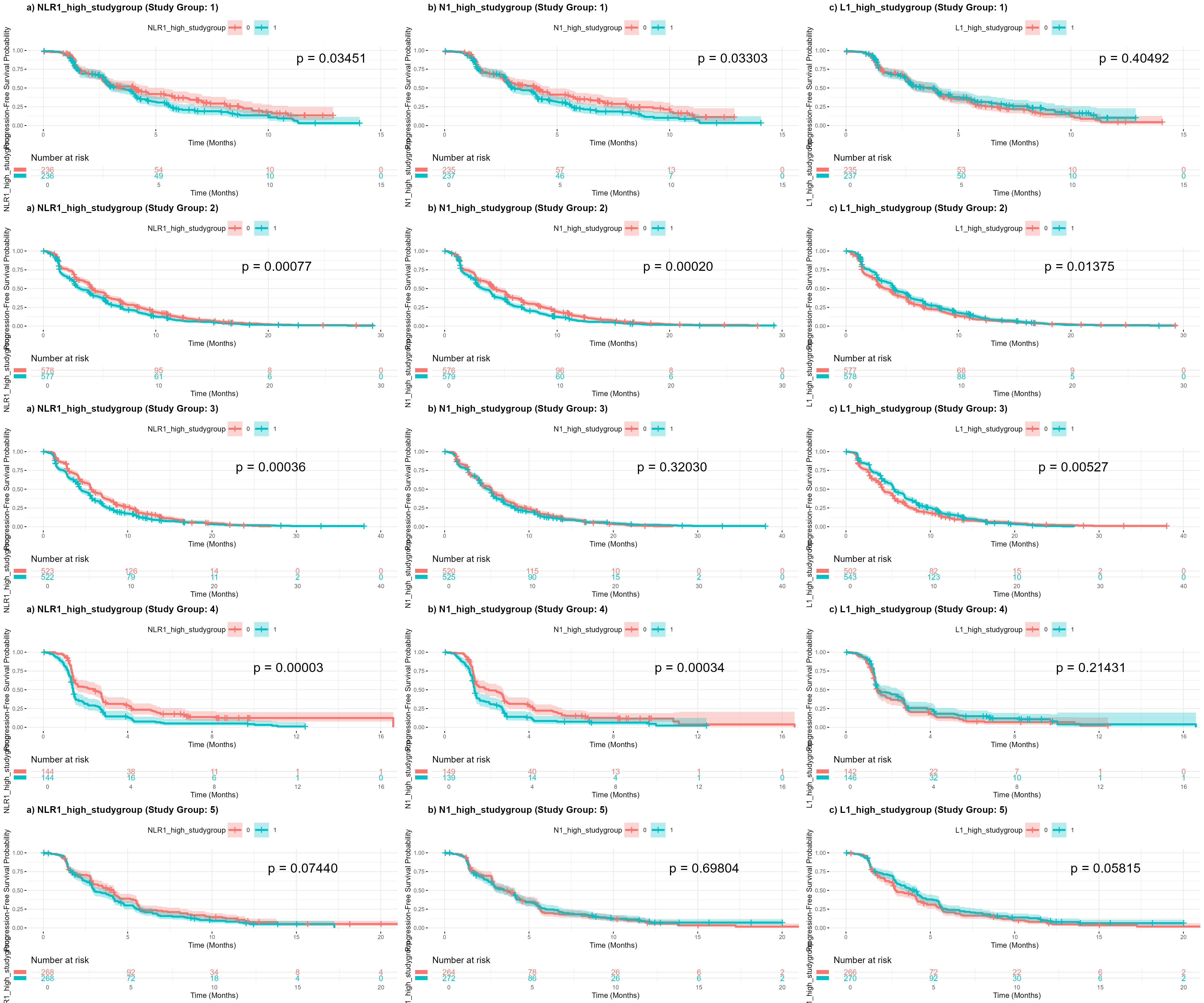


Supplementary Figure 4: Kaplan-Meier curves of progression-free survival according to high/low biomarkers and cancer type. Studygroup refers to the median calculated according to the median in each Study group. *NLR1: baseline lymphocyte-to-neutrophil ratio; N1: baseline neutrophil count; L1: baseline lymphocyte count.*


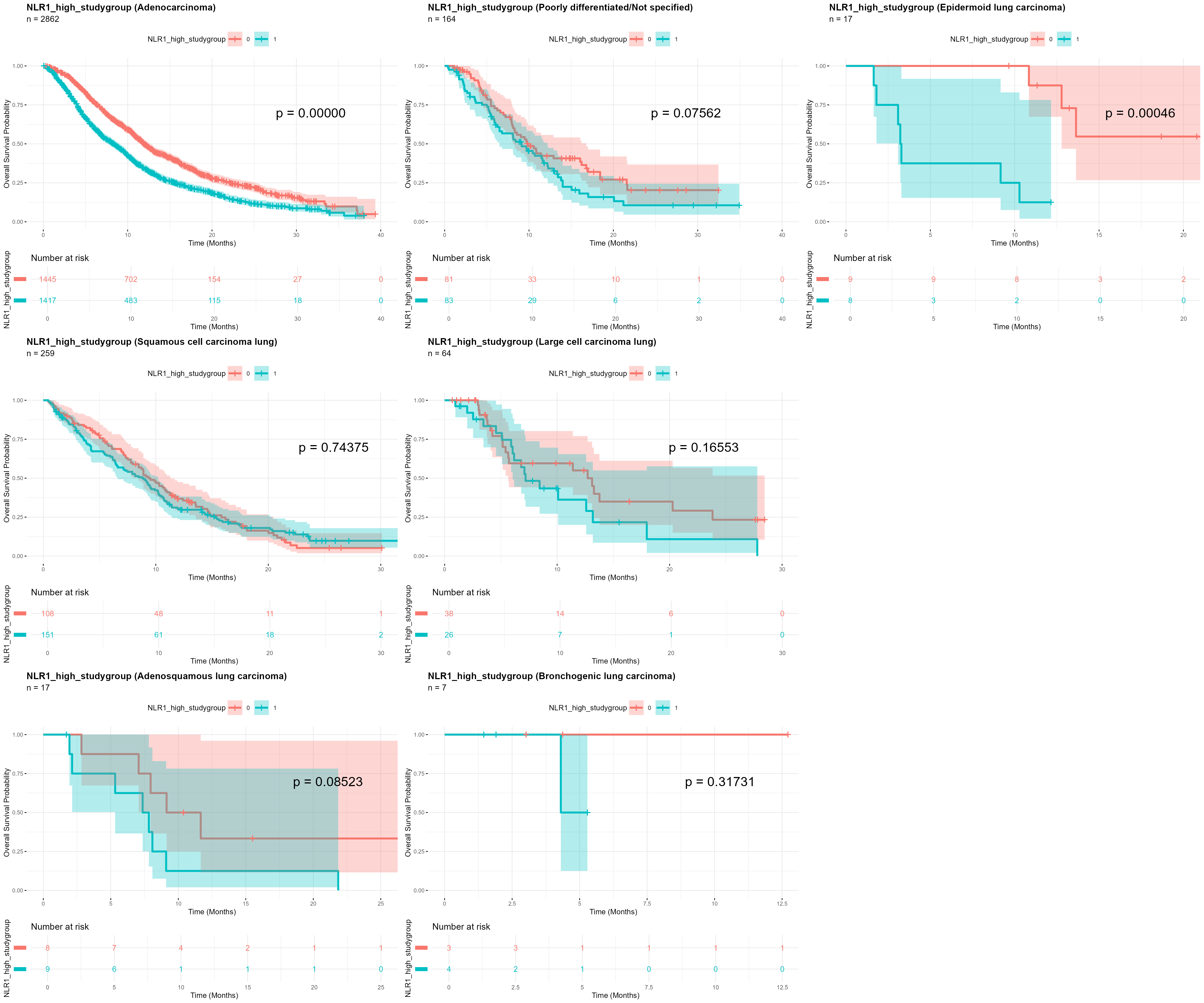


Supplementary Figure 5: Kaplan-Meier curves of overall survival according to high/low NLR1 (*baseline lymphocyte-to-neutrophil ratio)* and histological subtype. Studygroup refers to the median calculated according to the median in each Study group.


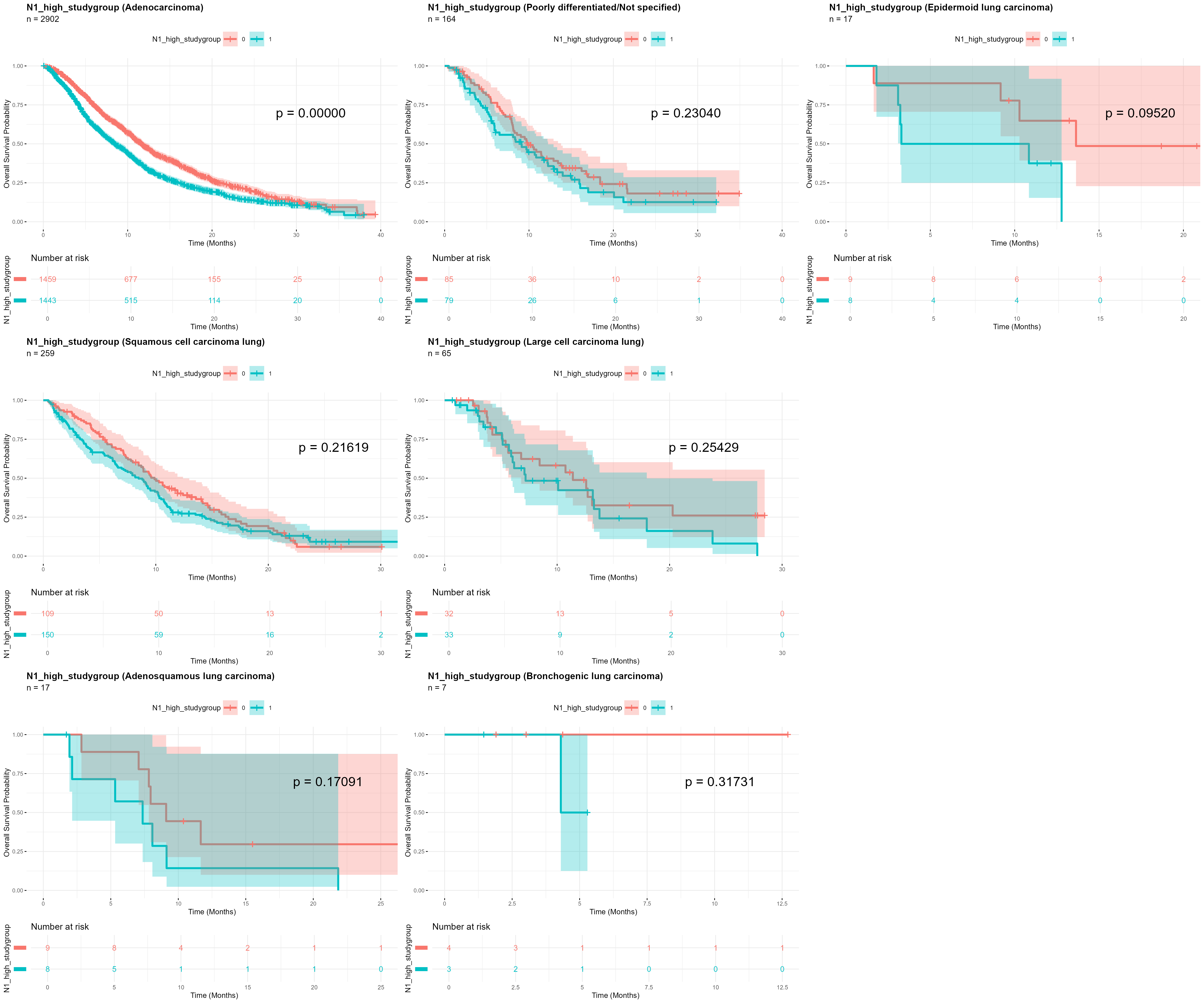


Supplementary Figure 6: Kaplan-Meier curves of overall survival according to high/low N1 (baseline neutrophil count) and histological subtype. Studygroup refers to the median calculated according to the median in each Study group.


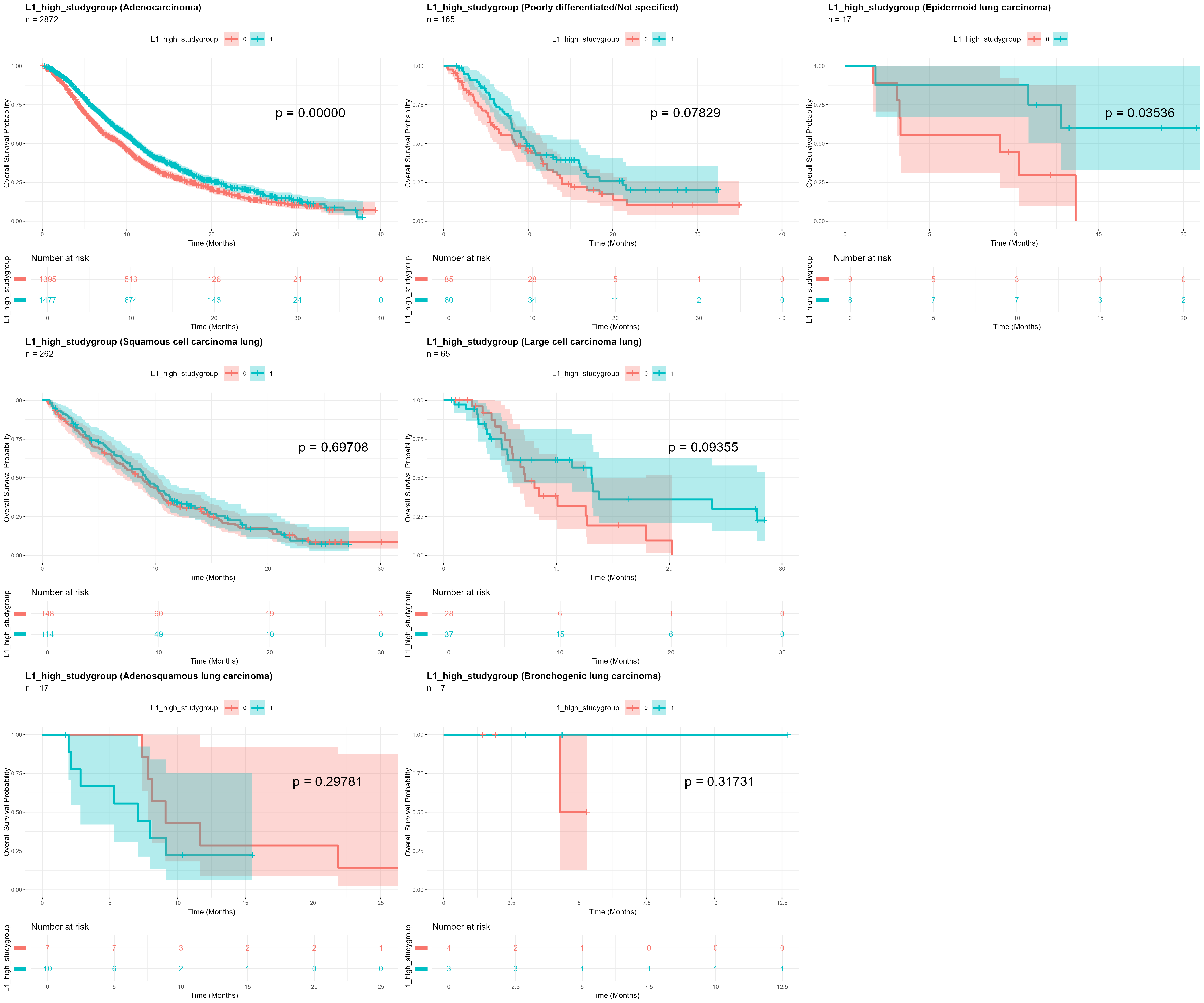


Supplementary Figure 7: Kaplan-Meier curves of overall survival according to high/low L1 (baseline lymphocyte count) and histological subtype. Studygroup refers to the median calculated according to the median in each Study group.


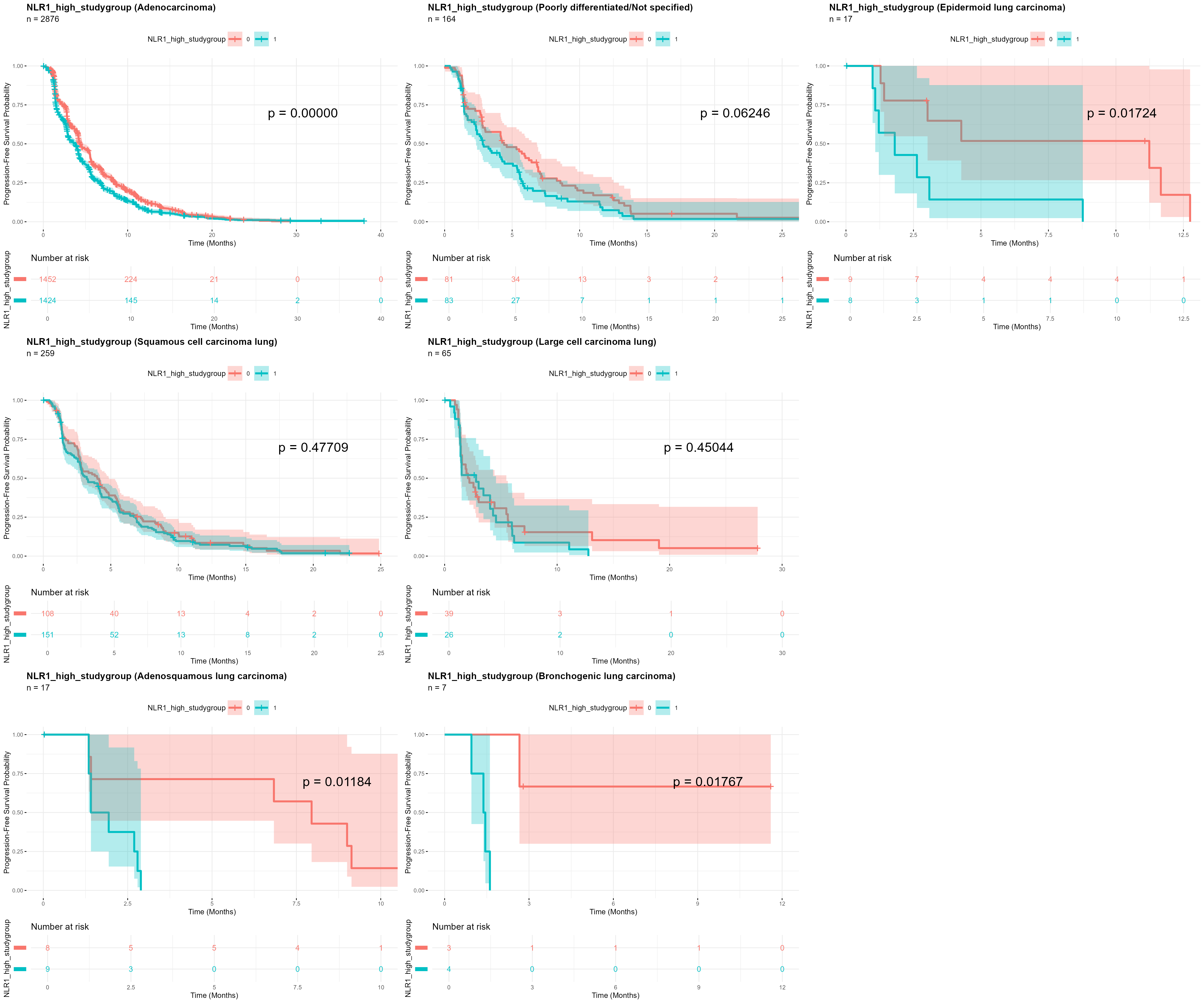


Supplementary Figure 8: Kaplan-Meier curves of progression-free survival according to high/low NLR1 (baseline lymphocyte-to-neutrophil ratio) and histological subtype. Studygroup refers to the median calculated according to the median in each Study group.


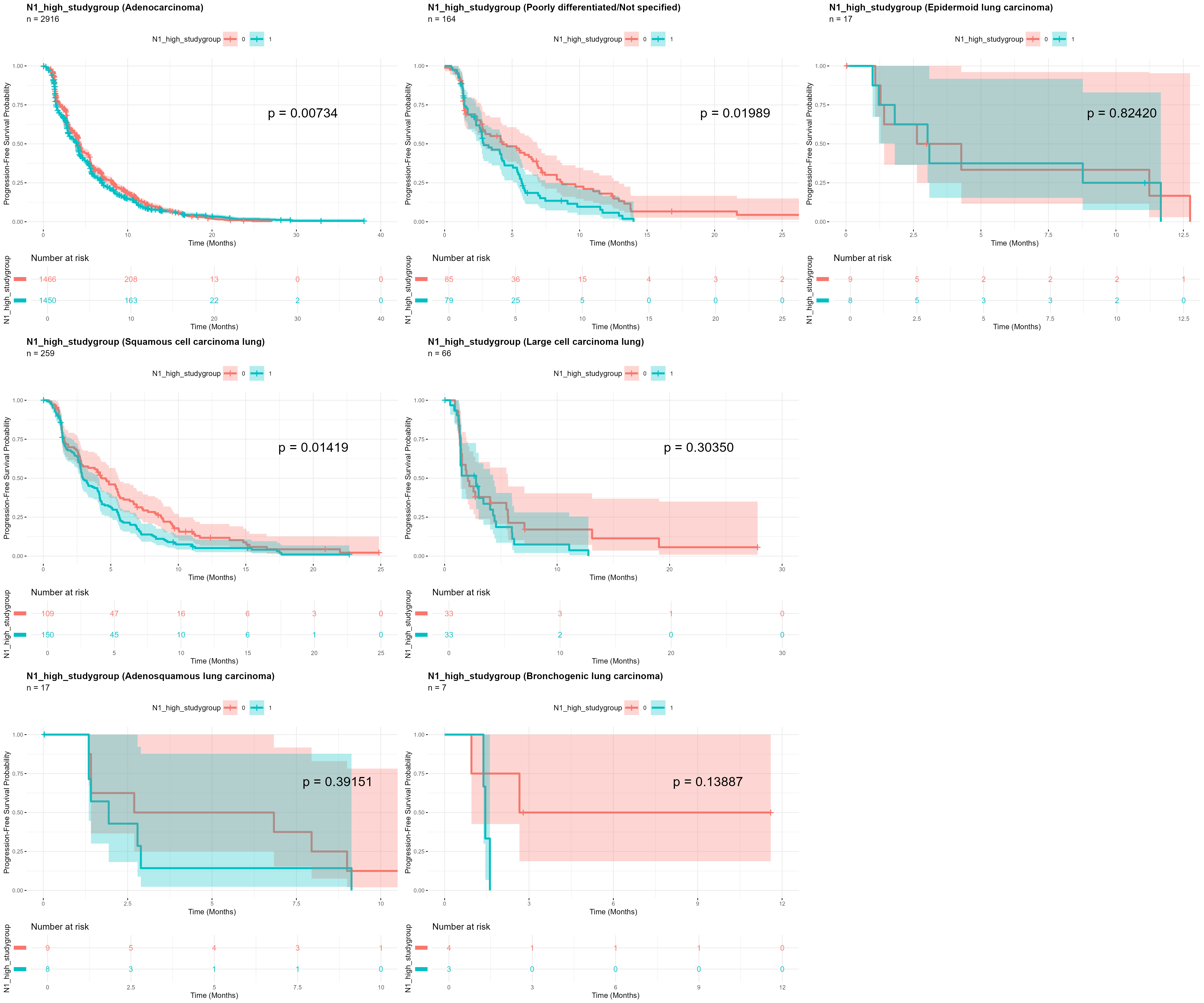


Supplementary Figure 9: Kaplan-Meier curves of progression-free survival according to high/low N1 (baseline neutrophil count) and histological subtype. Studygroup refers to the median calculated according to the median in each Study group.


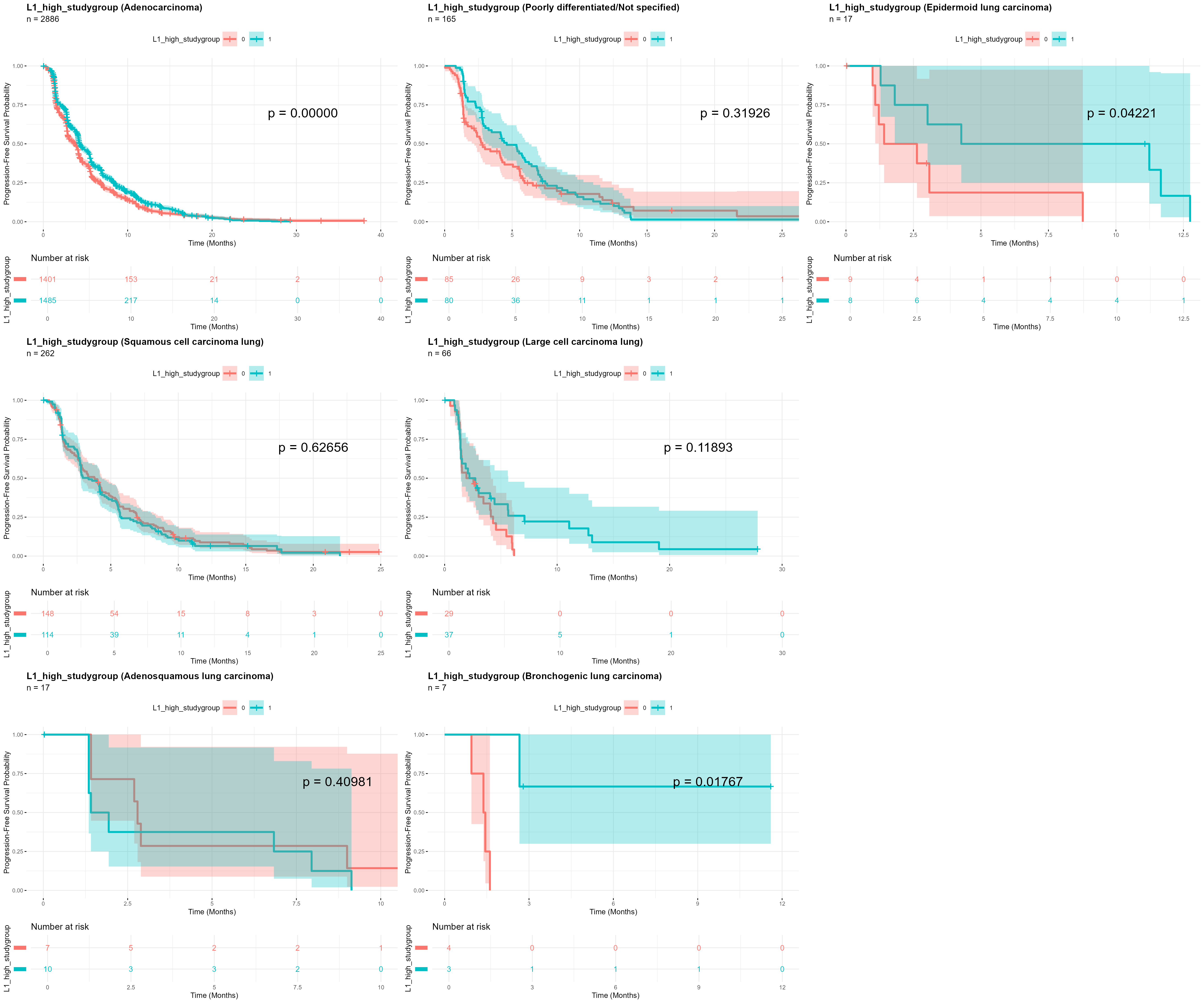


Supplementary Figure 10: Kaplan-Meier curves of progression-free survival according to high/low L1 (baseline lymphocyte count) and histological subtype. Studygroup refers to the median calculated according to the median in each Study group.

|  | Univariate analysis | |  | Multivariate analysis | |  |
| --- | --- | --- | --- | --- | --- | --- |
| Variable | *p*-value | HR | 95% CI HR | *p*-value | HR | 95% CI HR |
| Age ≥ 60y (Reference: < 60y | **0.012** | 0.913 | 0.850 – 0.980 | **0.005** | 0.889 | 0.819 – 0.966 |
| Race Black (Reference: Race White) | 0.767 | 0.964 | 0.755 – 1.231 | 0.117 | 0.814 | 0.630 – 1.053 |
| Race Other | **0.000** | 0.824 | 0.757 – 0.897 | **0.008** | 0.851 | 0.755 – 0.959 |
| Study 2 (Reference: Study 1) | **0.000** | 1.558 | 1.279 – 1.898 | **0.000** | 1.829 | 1.478 – 2.262 |
| Study 3 | **0.019** | 1.269 | 1.039 – 1.549 | **0.004** | 1.350 | 1.102 – 1.653 |
| Study 4 | **0.000** | 3.704 | 2.976 – 4.610 | **0.000** | 3.765 | 3.001 – 4.723 |
| Study 5 | **0.000** | 1.945 | 1.600 – 2.364 | **0.000** | 2.531 | 2.057 – 3.115 |
| Cancer Colorectal (Reference: Lung) | **0.003** | 0.867 | 0.788 – 0.953 | NA | NA | NA – NA |
| Cancer Gastric/GEJ | **0.000** | 1.476 | 1.359 – 1.603 | NA | NA | NA – NA |
| Treatment Active (Reference: Placebo) | **0.000** | 0.865 | 0.806 – 0.928 | **0.000** | 0.788 | 0.726 – 0.855 |
| Stage IV (Reference: IIIB | **0.014** | 2.379 | 1.188 – 4.763 | NA | NA | NA – NA |
| ECOG PS 1 (Reference: ECOG PS 0) | **0.000** | 1.471 | 1.369 – 1.581 | **0.000** | 1.534 | 1.382 – 1.703 |
| NLR1 | **0.000** | 1.031 | 1.024 – 1.038 | **0.000** | 1.035 | 1.028 – 1.042 |
| N1 | 0.074 | 1.000 | 1.000 – 1.001 | NA | NA | NA – NA |
| L1 | 0.375 | 1.001 | 0.999 – 1.003 | NA | NA | NA – NA |

Supplementary Table 3: Univariate COX hazard analysis of overall survival using baseline neutrophil-to-lymphocyte ratio (NLR1), neutrophil (N1) count and lymphocyte (L1) count as continuous variables. The variables that were significant in the univariate analysis were then tested in the multivariate analysis. Two models were used to test all variables with NLR1 and all variables with N1 and L1 to avoid multicollinearity issues. The multivariate results between both models did not vary significantly but the ones represented here at those with NLR1. Bold means statistically significant results with p < 0.05. *HR: hazard ratios; 95% CI HR: 95% confidence intervals of hazard ratios; NA: not available; y: years; ECOG PS: Eastern Cooperative Oncology Group Performance Status; NLR1: baseline lymphocyte-to-neutrophil ratio; N1: baseline neutrophil count; L1: baseline lymphocyte count.*

|  | Univariate analysis | |  | Multivariate analysis | |  |
| --- | --- | --- | --- | --- | --- | --- |
| Variable | *p*-value | HR | 95% CI HR | *p*-value | HR | 95% CI HR |
| Age ≥ 60y (Reference: < 60y | **0.012** | 0.913 | 0.850 – 0.980 | **0.008** | 0.893 | 0.822 – 0.971 |
| Race Black (Reference: Race White) | 0.767 | 0.964 | 0.755 – 1.231 | 0.439 | 0.904 | 0.699 – 1.168 |
| Race Other | **0.000** | 0.824 | 0.757 – 0.897 | **0.005** | 0.839 | 0.741 – 0.949 |
| Study 2 (Reference: Study 1) | **0.000** | 1.558 | 1.279 – 1.898 | **0.000** | 1.901 | 1.528 – 2.365 |
| Study 3 | **0.019** | 1.269 | 1.039 – 1.549 | **0.029** | 1.263 | 1.024 – 1.557 |
| Study 4 | **0.000** | 3.704 | 2.976 – 4.610 | **0.000** | 3.621 | 2.870 – 4.569 |
| Study 5 | **0.000** | 1.945 | 1.600 – 2.364 | **0.000** | 2.458 | 1.985 – 3.044 |
| Cancer Colorectal (Reference: Lung) | **0.003** | 0.867 | 0.788 – 0.953 | NA | NA | NA – NA |
| Cancer Gastric/GEJ | **0.000** | 1.476 | 1.359 – 1.603 | NA | NA | NA – NA |
| Treatment Active (Reference: Placebo) | **0.000** | 0.865 | 0.806 – 0.928 | **0.000** | 0.784 | 0.722 – 0.851 |
| Stage IV (Reference: IIIB | **0.014** | 2.379 | 1.188 – 4.763 | 0.282 | 1.484 | 0.723 – 3.047 |
| ECOG 1 (Reference: ECOG 0) | **0.000** | 1.471 | 1.369 – 1.581 | **0.000** | 1.527 | 1.374 – 1.696 |
| High NLR1 | **0.000** | 1.508 | 1.390 – 1.636 | **0.000** | 1.523 | 1.402 – 1.654 |
| High N1 | **0.000** | 1.390 | 1.282 – 1.507 | **0.000** | 1.396 | 1.285 – 1.516 |
| High L1 | **0.000** | 0.801 | 0.739 – 0.868 | **0.000** | 0.803 | 0.740 – 0.871 |

Supplementary Table 4: Univariate COX hazard analysis of overall survival using baseline high neutrophil-to-lymphocyte ratio (NLR1), high neutrophil (N1) count and high lymphocyte (L1) count as categorical variables. The variables that were significant in the univariate analysis were then tested in the multivariate analysis. Two models were used to test all variables with NLR1 and all variables with N1 and L1 to avoid multicollinearity issues. The multivariate results between both models did not vary significantly but the ones represented here at those with NLR1. Bold means statistically significant results with p < 0.05. *HR: hazard ratios; 95% CI HR: 95% confidence intervals of hazard ratios; NA: not available.*

|  | Univariate analysis | |  | Multivariate analysis | |  |
| --- | --- | --- | --- | --- | --- | --- |
| Variable | *p*-value | HR | 95% CI HR | *p*-value | HR | 95% CI HR |
| Age ≥ 60y (Reference: < 60y | **0.000** | 0.882 | 0.827 – 0.940 | **0.000** | 0.860 | 0.800 – 0.925 |
| Race Black (Reference: Race White) | 0.442 | 1.090 | 0.874 – 1.360 | NA | NA | NA – NA |
| Race Other | 0.253 | 0.957 | 0.888 – 1.032 | NA | NA | NA – NA |
| Study 2 (Reference: Study 1) | 0.201 | 0.920 | 0.809 – 1.046 | 0.355 | 0.934 | 0.807 – 1.080 |
| Study 3 | **0.000** | 0.721 | 0.632 – 0.822 | **0.000** | 0.704 | 0.616 – 0.805 |
| Study 4 | **0.000** | 1.981 | 1.688 – 2.324 | **0.000** | 2.128 | 1.796 – 2.521 |
| Study 5 | 0.351 | 0.941 | 0.828 – 1.069 | 0.789 | 0.980 | 0.847 – 1.135 |
| Cancer Colorectal (Reference: Lung) | **0.000** | 0.773 | 0.713 – 0.839 | NA | NA | NA – NA |
| Cancer Gastric/GEJ | **0.001** | 1.130 | 1.050 – 1.215 | NA | NA | NA – NA |
| Treatment Active (Reference: Placebo) | **0.000** | 0.755 | 0.709 – 0.804 | **0.000** | 0.709 | 0.659 – 0.763 |
| Stage IV (Reference: IIIB | 0.107 | 1.412 | 0.929 – 2.147 | NA | NA | NA – NA |
| ECOG 1 (Reference: ECOG 0) | **0.000** | 1.204 | 1.130 – 1.284 | **0.000** | 1.245 | 1.136 – 1.365 |
| NLR1 | **0.000** | 1.022 | 1.016 – 1.029 | **0.000** | 1.021 | 1.015 – 1.028 |
| N1 | **0.003** | 1.001 | 1.000 – 1.001 | **0.007** | 1.002 | 1.000 – 1.003 |
| L1 | 0.194 | 1.001 | 0.999 – 1.003 | NA | NA | NA – NA |

Supplementary Table 5: Univariate COX hazard analysis of progression-free survival using baseline neutrophil-to-lymphocyte ratio (NLR1), neutrophil (N1) count and lymphocyte (L1) count as continuous variables. The variables that were significant in the univariate analysis were then tested in the multivariate analysis. Two models were used to test all variables with NLR1 and all variables with N1 and L1 to avoid multicollinearity issues. The multivariate results between both models did not vary significantly but the ones represented here at those with NLR1. Bold means statistically significant results with p < 0.05. *HR: hazard ratios; 95% CI HR: 95% confidence intervals of hazard ratios; NA: not available.*

|  | Univariate analysis | |  | Multivariate analysis | |  |
| --- | --- | --- | --- | --- | --- | --- |
| Variable | *p*-value | HR | 95% CI HR | *p*-value | HR | 95% CI HR |
| Age ≥ 60y (Reference: < 60y | **0.000** | 0.882 | 0.827 – 0.940 | **0.000** | 0.861 | 0.801 – 0.926 |
| Race Black (Reference: Race White) | 0.442 | 1.090 | 0.874 – 1.360 | NA | NA | NA – NA |
| Race Other | 0.253 | 0.957 | 0.888 – 1.032 | NA | NA | NA – NA |
| Study 2 (Reference: Study 1) | 0.201 | 0.920 | 0.809 – 1.046 | 0.725 | 0.974 | 0.844 – 1.126 |
| Study 3 | **0.000** | 0.721 | 0.632 – 0.822 | **0.000** | 0.690 | 0.604 – 0.789 |
| Study 4 | **0.000** | 1.981 | 1.688 – 2.324 | **0.000** | 2.118 | 1.788 – 2.509 |
| Study 5 | **0.351** | 0.941 | 0.828 – 1.069 | 0.712 | 0.973 | 0.840 – 1.126 |
| Cancer Colorectal (Reference: Lung) | **0.000** | 0.773 | 0.713 – 0.839 | NA | NA | NA – NA |
| Cancer Gastric/GEJ | **0.001** | 1.130 | 1.050 – 1.215 | NA | NA | NA – NA |
| Treatment Active (Reference: Placebo) | **0.000** | 0.755 | 0.709 – 0.804 | **0.000** | 0.707 | 0.657 – 0.761 |
| Stage IV (Reference: IIIB | 0.107 | 1.412 | 0.929 – 2.147 | NA | NA | NA – NA |
| ECOG 1 (Reference: ECOG 0) | **0.000** | 1.204 | 1.130 – 1.284 | **0.000** | 1.245 | 1.137 – 1.365 |
| High NLR1 | **0.000** | 1.261 | 1.174 – 1.355 | **0.000** | 1.256 | 1.169 – 1.350 |
| High N1 | **0.000** | 1.154 | 1.074 – 1.239 | **0.000** | 1.178 | 1.096 – 1.267 |
| High L1 | **0.000** | 0.848 | 0.789 – 0.911 | **0.000** | 0.866 | 0.806 – 0.931 |

Supplementary Table 6: Univariate COX hazard analysis of progression-free survival using baseline high neutrophil-to-lymphocyte ratio (NLR1), high neutrophil (N1) count and high lymphocyte (L1) count as categorical variables. The variables that were significant in the univariate analysis were then tested in the multivariate analysis. Two models were used to test all variables with NLR1 and all variables with N1 and L1 to avoid multicollinearity issues. The multivariate results between both models did not vary significantly but the ones represented here at those with NLR1. Bold means statistically significant results with p < 0.05. *HR: hazard ratios; 95% CI HR: 95% confidence intervals of hazard ratios; NA: not available.*

|  | High NLR1 |  |  | High N1 |  |  | | High L1 | | | | |  | | | | |  |  |  |  |  |
| --- | --- | --- | --- | --- | --- | --- | --- | --- | --- | --- | --- | --- | --- | --- | --- | --- | --- | --- | --- | --- | --- | --- |
|  | *p*-val. | HR | 95% CI | *p*-val. | HR | 95% CI | | *p*-val. | | | HR | | | | | 95% CI | | | | | | |
| Age |  |  |  |  |  |  |  | |  | | | | |  | | | | | | |  |  |
| < 60 years | **0.000** | **1.596** | 1.410 – 1.807 | **0.000** | **1.441** | 1.274 – 1.628 | | **0.000** | | **0.768** | | | | | 0.680 – 0.868 | | | | |  |  |  |
| ≥ 60 years | **0.000** | 1.443 | 1.295 – 1.608 | **0.000** | 1.349 | 1.211 – 1.502 | | **0.000** | | 0.822 | | | | | 0.738 – 0.916 | | | | | | |  |
| Race |  |  |  |  |  |  | |  | |  | | | | |  | | | | | | |  |
| White | **0.000** | 1.489 | 1.362 – 1.629 | **0.000** | 1.364 | 1.248 – 1.491 | | **0.000** | | **0.785** | | | | | 0.718 – 0.857 | | | | | | |  |
| Black | **0.044** | **1.708** | 1.015 – 2.875 | 0.150 | 1.465 | 0.871 – 2.465 | | 0.723 | | 0.914 | | | | | 0.557 – 1.501 | | | | | | |  |
| Other | **0.001** | 1.434 | 1.150 – 1.789 | **0.002** | **1.394** | 1.124 – 1.728 | | 0.283 | | 0.888 | | | | | 0.715 – 1.103 | | | | | | |  |
| Stage |  |  |  |  |  |  | |  | |  | | | | |  | | | | | | |  |
| Stage III B | 0.442 | 1.895 | 0.372 – 9.655 | 0.419 | 1.967 | 0.381 – 10.153 | | 0.183 | | 0.237 | | | | | 0.029 – 1.972 | | | | | | |  |
| Stage IV | **0.000** | **1.514** | 1.394 – 1.643 | **0.000** | **1.393** | 1.284 – 1.511 | | **0.000** | | **0.801** | | | | | 0.738 – 0.869 | | | | | | |  |
| Cancer type (Study group) | |  |  |  |  |  |  | | | | |  | | | | |  | |  |  |  |  |
| Lung | **0.000** | 1.482 | 1.302 – 1.687 | **0.000** | **1.441** | 1.266 – 1.640 | | **0.000** | | **0.761** | | | | | 0.669 – 0.856 | | | | | | |  |
| Colorectal | **0.000** | 1.568 | 1.357 – 1.811 | **0.000** | 1.428 | 1.237 – 1.648 | | 0.050 | | 0.867 | | | | | 0.751 – 1.000 | | | | | | |  |
| Gastric or GEJ | **0.000** | **1.591** | 1.366 – 1.853 | **0.000** | 1.342 | 1.156 – 1.557 | | **0.002** | | 0.789 | | | | | 0.678 – 0.919 | | | | | | |  |
| Study group |  |  |  |  |  |  | |  | |  | | | | |  | | | | | | |  |
| 1 | **0.000** | 2.213 | 1.488 – 3.291 | **0.000** | **2.024** | 1.372 – 2.985 | | 0.060 | | 0.698 | | | | | 0.480 – 1.015 | | | | | | |  |
| 2 | **0.000** | 1.416 | 1.234 –1.626 | **0.000** | 1.378 | 1.201 – 1.582 | | **0.000** | | **0.767** | | | | | 0.669 – 0.880 | | | | | | |  |
| 3 | **0.000** | 1.568 | 1.357 – 1.811 | **0.000** | 1.428 | 1.237 – 1.648 | | 0.050 | | 0.867 | | | | | 0.751 – 1.000 | | | | | | |  |
| 4 | **0.000** | **1.816** | 1.396 – 2.362 | **0.000** | 1.564 | 1.219 – 2.008 | | 0.053 | | 0.773 | | | | | 0.596 – 1.003 | | | | | | |  |
| 5 | **0.000** | 1.506 | 1.249 – 1.817 | **0.016** | 1.258 | 1.044 – 1.516 | | **0.017** | | 0.796 | | | | | 0.660 – 0.961 | | | | | | |  |
| Treatment group |  |  |  |  |  |  | |  | |  | | | | |  | | | | | | |  |
| Placebo | **0.000** | **1.578** | 1.404 – 1.773 | **0.000** | 1.355 | 1.208 – 1.521 | | **0.000** | | **0.802** | | | | | 0.714 – 0.900 | | | | | | |  |
| Active | **0.000** | 1.441 | 1.286 – 1.615 | **0.000** | **1.429** | 1.275 – 1.601 | | **0.000** | | 0.809 | | | | | 0.723 – 0.907 | | | | | | |  |
| Baseline ECOG PS |  |  |  |  |  |  | |  | |  | | | | |  | | | | | | |  |
| 0 | **0.000** | 1.425 | 1.283 – 1.581 | **0.000** | 1.339 | 1.207 – 1.486 | | **0.000** | | **0.793** | | | | | 0.715 – 0.880 | | | | | | |  |
| 1 | **0.000** | **1.600** | 1.403 – 1.824 | **0.000** | **1.404** | 1.235 – 1.598 | | **0.003** | | 0.824 | | | | | 0.724 – 0.938 | | | | | | |  |
| Cancer type (cancer type) | |  |  |  |  |  |  | | | | |  | | | | |  | |  |  |  |  |
| Lung | **0.000** | 1.519 | 1.333 – 1.730 | **0.000** | **1.445** | 1.270 – 1.645 | | **0.000** | | **0.751** | | | | | 0.660 – 0.855 | | | | | | |  |
| Colorectal | **0.000** | **1.568** | 1.357 – 1.811 | **0.000** | 1.428 | 1.237 – 1.648 | | 0.050 | | 0.867 | | | | | 0.751 – 1.000 | | | | | | |  |
| Gastric or GEJ | **0.000** | 1.488 | 1.278 – 1.734 | **0.000** | 1.324 | 1.140 – 1.536 | | **0.007** | | 0.811 | | | | | 0.697 – 0.945 | | | | | | |  |

Supplementary Table 7: Multivariate COX hazard analysis of the variables that were significant in the multivariate analysis divided into subsets. We show the hazard ratios, 95% confidence interval and p-values of the subsets in relation to overall survival of baseline high neutrophil-to-lymphocyte ratio (NLR1), high neutrophil (N1) count and high lymphocyte (L1) count according to the median of each study group except for cancer type where high was categorized according to the median of the cancer type. Bold means statistically significant results with p < 0.05. *p*-val.: *p*-value; *HR: hazard ratios; 95% CI HR: 95% confidence intervals of hazard ratios; NA: not available. ECOG PS: Eastern Cooperative Oncology Group Performance Status.*

| Biomarker | *p*-value AUCs overall survival | *p*-value AUCs progression-free survival |
| --- | --- | --- |
| N1 | **0.000** | **0.004** |
| L1 | NA | NA |
| NLR1 | 0.203 | **0.017** |
| N2 | **0.001** | 0.186 |
| L2 | 0.074 | 0.088 |
| NLR2 | **0.000** | 0.127 |
| N3 | **0.000** | 0.654 |
| L3 | 0.223 | 0.186 |
| NLR3 | **0.033** | 0.684 |
| Percen_NLR1_NLR2 | **0.000** | 0.103 |
| Percen_NLR1_NLR3 | **0.070** | 0.103 |

Supplementary Table 8: *p*-values of the areas under the curve (AUC) of the biomarkers with the highest AUC (L1) vs. the other biomarkers for both overall survival and progression-free survival. Bold indicates *p-values* < 0.05. *NLR1: baseline neutrophil-to-lymphocyte count; N1: baseline neutrophil count; L1: baseline lymphocyte count; NLR2: neutrophil-to-lymphocyte count at 3 weeks; N2: neutrophil count at 3 weeks; L2: lymphocyte count at 3 weeks; NLR3: neutrophil-to-lymphocyte count at 6 weeks; N3: neutrophil count at 6 weeks; L3: lymphocyte count at 6 weeks; PercenNLR1_NLR2: percentage of the change from NLR1 to NLR2 over the baseline NLR1; PercenNLR2_NLR3: percentage of the change from NLR1 to NLR3 over the baseline NLR1.*

| Biomarker | Outcome | Cutoff_Type | Cutoff | Sensitivity | Specificity | Accuracy | PPV | NPV | AUC |
| --- | --- | --- | --- | --- | --- | --- | --- | --- | --- |
| **NLR1** | **OS** | **Median** | **3.33** | **0.51** | **0.52** | **0.51** | **0.87** | **0.15** | **0.51** |
| NLR1 | OS | Youden | 3.63 | 0.46 | 0.56 | 0.47 | 0.87 | 0.14 | 0.51 |
| NLR1 | OS | Balanced | 3.59 | 0.47 | 0.56 | 0.48 | 0.87 | 0.14 | 0.51 |
| N1 | OS | Median | 4.86 | 0.49 | 0.46 | 0.49 | 0.85 | 0.13 | 0.52 |
| **N1** | **OS** | **Youden** | **6.66** | **0.28** | **0.69** | **0.34** | **0.85** | **0.14** | **0.49** |
| N1 | OS | Balanced | 4.90 | 0.49 | 0.47 | 0.49 | 0.85 | 0.13 | 0.52 |
| **L1** | **OS** | **Median** | **1.41** | **0.49** | **0.45** | **0.49** | **0.85** | **0.13** | **0.53** |
| L1 | OS | Youden | 1.15 | 0.66 | 0.31 | 0.61 | 0.85 | 0.13 | 0.48 |
| L1 | OS | Balanced | 1.52 | 0.43 | 0.49 | 0.44 | 0.84 | 0.12 | 0.54 |
| **NLR1** | **PFS** | **Median** | **3.33** | **0.50** | **0.51** | **0.50** | **0.86** | **0.14** | **0.50** |
| NLR1 | PFS | Youden | 6.18 | 0.23 | 0.82 | 0.31 | 0.89 | 0.15 | 0.53 |
| NLR1 | PFS | Balanced | 3.56 | 0.48 | 0.58 | 0.49 | 0.87 | 0.15 | 0.53 |
| N1 | PFS | Median | 4.87 | 0.50 | 0.47 | 0.49 | 0.85 | 0.13 | 0.51 |
| **N1** | **PFS** | **Youden** | **3.66** | **0.70** | **0.26** | **0.64** | **0.85** | **0.12** | **0.48** |
| N1 | PFS | Balanced | 4.45 | 0.55 | 0.39 | 0.53 | 0.85 | 0.12 | 0.47 |
| **L1** | **PFS** | **Median** | **1.41** | **0.49** | **0.43** | **0.48** | **0.84** | **0.12** | **0.54** |
| L1 | PFS | Youden | 1.31 | 0.55 | 0.38 | 0.52 | 0.84 | 0.12 | 0.46 |
| L1 | PFS | Balanced | 1.36 | 0.53 | 0.40 | 0.51 | 0.84 | 0.12 | 0.46 |

Supplementary Table 9: Optimal cut-off values fo for overall survival and progression-free survival calculated according to three methods: the Median, Youden’s index and a Balanced cut-off. In bold is represented the best cut-off value prioritizing sensitivity, positive predictive value and area under the curve. *PPV: positive predictive value; NPV: negative predictive value; AUC: area under the curve;* *OS: overall survival; PFS: progression-free survival;* *NLR1: baseline neutrophil-to-lymphocyte count; N1: baseline neutrophil count; L1: baseline lymphocyte count.*

| Subset | AUC | n | *p*-value |
| --- | --- | --- | --- |
| NLR1 OS |  |  |  |
| < 60 years + Black + Study 5 | 1 | 12 | 0.010 |
| N1 OS |  |  |  |
| < 60 years + Black + Study 5 | 1 | 12 | 0.033 |
| L1 OS |  |  |  |
| < 60 years + Black + Study 5 | 1 | 12 | 0.039 |
| NLR1 PFS |  |  |  |
| Black + Gastric/GEJ + ECOG PS 0 | 1 | 9 | 0.006 |
| Black + Study 5 + ECOG PS 0 | 1 | 8 | 0.028 |
| N1 PFS |  |  |  |
| Black + Study 5 + Active treatment | 1 | 12 | 0.026 |
| Black + Gastric/GEJ + ECOG PS 0 | 1 | 9 | 0.005 |
| Black + Study 5 + ECOG PS 0 | 1 | 8 | 0.024 |
| L1 PFS |  |  |  |
| – | – | – | – |

Supplementary Table 10: Subsets of patients in which the biomarkers had an area under the curve (AUC) of 1. *OS: overall survival; NLR1: baseline neutrophil-to-lymphocyte count; N1: baseline neutrophil count; L1: baseline lymphocyte count; PFS: progression-free survival; ECOG PS: Eastern Cooperative Oncology Group Performance Status.*
